# Metabolic and bariatric surgery and male endocrine and reproductive health: a GRADE-assessed meta-analysis

**DOI:** 10.3389/fnut.2026.1775651

**Published:** 2026-03-16

**Authors:** Ruihui Zhu, Lili Li

**Affiliations:** 1 Department of Clinical Laboratory, Sir Run Run Shaw Hospital, Zhejiang University School of Medicine, Hangzhou, Zhejiang, China; 2 Department of Clinical Laboratory, Huzhou Central Hospital, Affiliated Central Hospital of Huzhou University, Huzhou, Zhejiang, China

**Keywords:** bariatric surgery, male reproductive function, obesity, semen quality, sex hormones, sexual function

## Abstract

**Background:**

The negative impact of obesity on male fertility and sexual health is well-established. Although metabolic and bariatric surgery (MBS) offers a powerful solution for severe obesity, its impact on the male androgens, semen quality, and sexual function are not yet fully understood.

**Methods:**

A comprehensive systematic search of the literature was conducted in PubMed, Embase, the Cochrane Library, and Web of Science for relevant studies published up to July 2024. Data about reproductive hormones, semen parameters, and sexual function in men with obesity undergoing bariatric surgery was extracted. For data synthesis, weighted mean differences (WMDs) and their 95% confidence intervals (CIs) were calculated using random- or fixed-effects models, as appropriate.

**Results:**

Fifty-nine studies with 60 arms were included in our meta-analysis. Metabolic and bariatric surgery indicated elevated levels of total testosterone (TT) (WMD: 5.46 nmol/L, *p* < 0.001; WMD: 6.58 nmol/L, *p* < 0.001; and WMD: 8.02 nmol/L, *p* < 0.001), sex hormone-binding globulin (SHBG) (WMD: 14.56 nmol/L, *p* < 0.001; WMD: 18.08 nmol/L, *p* < 0.001; and WMD: 23.64 nmol/L, *p* < 0.001), international index of erectile function (IIEF) (WMD: 9.36, *p* < 0.001), and diminished levels of PRL (WMD: −76.48 mIU/L, *p* = 0.003) at all intervals. Moreover, increased levels of free testosterone (FT) at 6–9 month (WMD: 66.61 nmol/L, *p* < 0.001), and >12 months (WMD: 78.94 nmol/L; *p* < 0.001) were observed pos-operative state among obese men. Also, decreased levels of estradiol (E_2_) at 6–9 month (WMD: −14.52 nmol/L, *p* = 0.005), and ≥12 months (WMD: −11.63 nmol/L; *p* < 0.001) was seen after bariatric surgery.

**Conclusion:**

Overally, this meta-analysis illustrated that bariatric surgery improves the hormonal profile and sexual function in obese men profoundly. These findings introduce bariatric surgery as an effective therapeutic strategy for weight loss management and male reproductive health improvement.

## 1 Introduction

Obesity is a rapidly growing global epidemic with severe systemic consequences (1). Epidemiological studies estimate that by 2035, over 1.5 billion adults will have obesity, presenting a healthcare challenge (2). Annually, obesity is linked to over 4.72 million deaths worldwide and is a major risk factor for comorbidities including metabolic disease (3). Beyond this, its detrimental impact extends to reproductive health too (4). The pathophysiological hallmarks of obesity such as chronic inflammation and endocrine dysregulation impair reproductive function, contributing to male subfertility and female disorders like PCOS (4–6). Previous studies illustrated profound influence of adiposity on reproductive endocrine health.

Metabolic and bariatric surgery (MBS), primarily through procedures like Roux-en-Y gastric bypass (GB) and sleeve gastrectomy (SG), has become a cornerstone treatment for severe obesity (7). These interventions induce profound weight loss and ameliorate metabolic disorders via mechanisms such as gastric restriction, malabsorption, and altered gut hormone secretion (8, 9). Despite these well-documented systemic benefits, the specific impact of MBS on male reproductive function is an area of ongoing debate (10). While a body of evidence suggests potential benefits including increased serum testosterone, improved semen parameters, and enhanced sexual function, other studies report concerning outcomes, such as postoperative hormonal imbalance, worsening semen quality, and declines in sexual function scores (11, 12).

Given the expanding use of MBS and persistent uncertainties regarding its effects on male reproduction, an updated evidence synthesis is urgently needed. Prior meta-analyses, while indicating potential benefits, are limited by methodological shortcomings and are challenged by newer studies showing variable outcomes dependent on surgical technique and follow-up time. To address this, we conducted a systematic review and meta-analysis of recent evidence to comprehensively evaluate the impact of BS on male reproductive hormones, sexual function, and semen parameters. This work provides a refined evidence base to guide the clinical management of obese men with reproductive dysfunction and to inform relevant public health strategies.

## 2 Materials and methods

Our meta-analysis was conducted using the Preferred Reporting Items for Systematic Reviews and Meta-Analyses (PRISMA) (13). The study protocol was registered with the International Prospective Register of Systematic Reviews (PROSPERO: CRD420261309015).

### 2.1 Search strategy

A comprehensive systematic search was conducted through PubMed, Embase, Cochrane Library, and Web of Science databases from inception to July 2024. The systematic search using Medical Subject Headings (MeSH) is as below: “bariatric surgical procedure”, “weight reduction surgery”, “obesity operation,” “gonadal hormones,” “sex hormones”, “gonadal steroid hormones”, “semen quality analyses”, “international index of erectile function (IIEF)”, as well as “seminal plasma analysis”. In addition, hand-searching was done to avoid missing any relevant studies. The search strategy is available in Supplementary Table 1.

### 2.2 Eligibility criteria and extraction process

The screening process was completed by two investigators independently and any disagreement was discussed. The inclusion criteria were as follow: Participants: participants were obese male patients undergoing MBS; Outcomes: TT, E_2_, FT, luteinizing hormone (LH), follicle-stimulating hormone (FSH), SHBG, prolactin (PRL), sperm concentration (SC), total sperm count (TSC), semen volume (SV), percentages of morphologically normal, total motile sperm, and IIEF-5; Study design: observational cohort studies. While, studies with following criteria were excluded: animal or cellular studies, reviews, grey literature, including: ClinicalTrials.gov, WHO International Clinical Trials Registry Platform (ICTRP), and conference abstracts, study protocols, case reports letters, and incomplete data or serious methodological errors.

Based on eligibility criteria following data were extracted: first author, publication year, location, baseline characteristics of participants, type of MBS, follow-up duration, and reported outcomes.

### 2.3 Quality assessment

The methodological quality of included studies was assessed using the Newcastle-Ottawa Scale (NOS), which comprises eight items across three domains: selection, comparability, and exposure (14). The NOS yields a score ranging from 0 to 9, with a score of 6 or higher generally indicating high-quality studies, and a score of 5 or lower reflecting lower methodological quality. Following the GRADE approach, we evaluated the certainty of the evidence for each outcome based on five standard domains: risk of bias, inconsistency, indirectness, imprecision, and publication bias.

### 2.4 Statistical analysis

Statistical analysis was conducted using STATA software, version 18. The relationship between MBS and male reproductive function was evaluated by calculating WMDs alongside their 95% CIs. To quantify heterogeneity across studies, we employed the Cochrane *Q* test and the *I*^2^ statistic. Significant heterogeneity was predefined as an *I*^2^ statistic exceeding 50%. Based on this assessment, a random-effects model was applied when substantial heterogeneity was present; otherwise, a fixed-effects model was used. The robustness of the pooled results was examined through sensitivity analysis. We investigated potential sources of heterogeneity via subgroup analyses, stratified by surgical procedure and duration of follow-up. To explore potential sources of heterogeneity, we performed meta-regression analyses examining the impact of surgical type, follow-up duration, baseline BMI, and study quality (NOS score) as covariates. For outcomes reported in more than 10 studies, publication bias was assessed visually using funnel plots and quantitatively evaluated with Egger’s test, where a *p*-value <0.05 was considered indicative of significant bias (15).

## 3 Results

### 3.1 Study selection and characteristics

**Figure 1:**
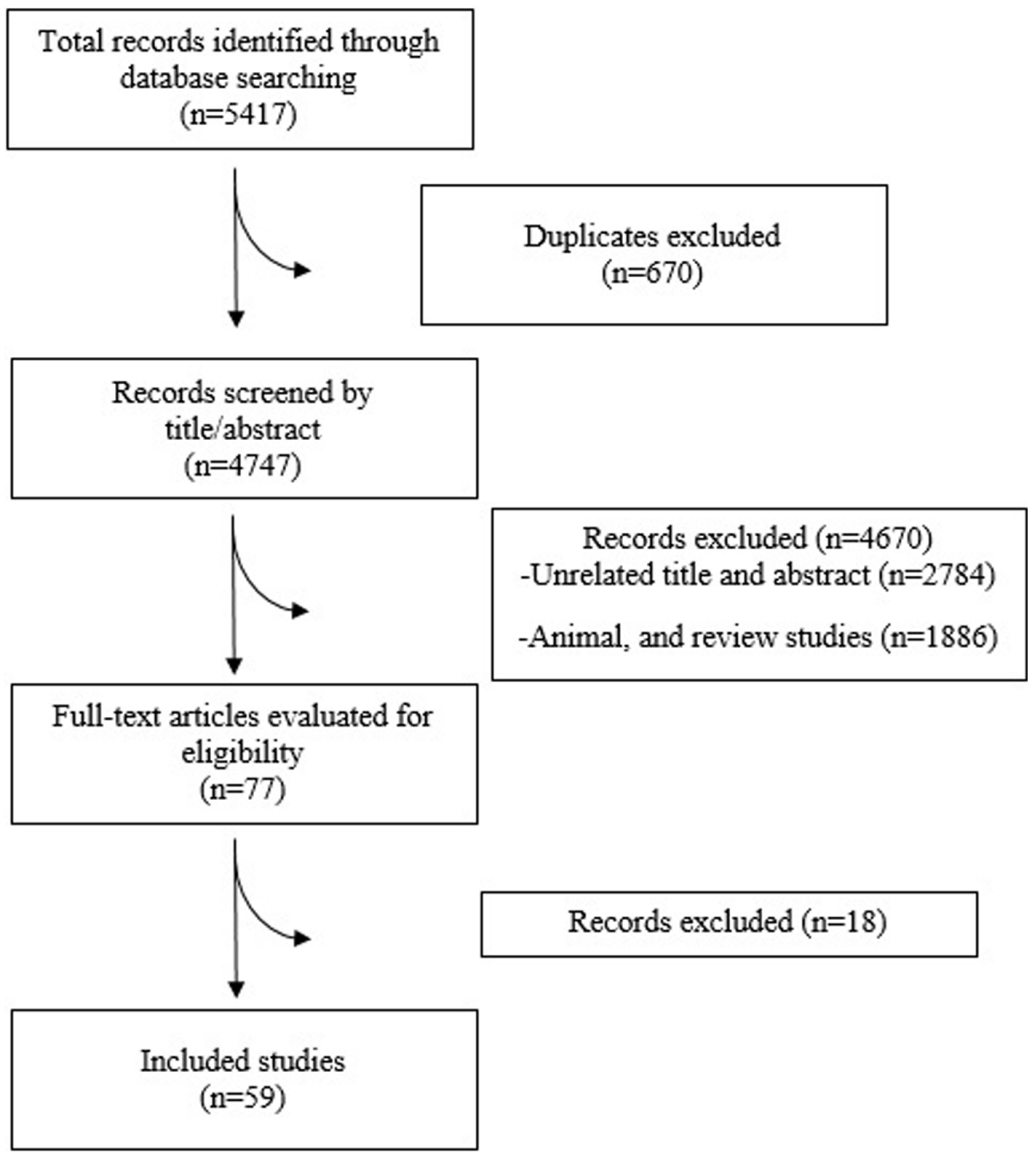
Flow diagram for selection and screening of studies.

The systematic search retrieved 5,417 records. Then, 670 studies were detected as duplicates and were removed. The 4,747 remained studies were screened using title and abstract. Likewise, the remained studies were checked for their eligibility for this study and we assessed their full-text. Finally, 59 studies (comprising 60 study arms) met our inclusion criteria and were included in this study. The study selection flow diagram is provided in the Figure 1.

The baseline characteristics of the included studies are presented in the Table 1. This meta-analysis included 2,401 men that were obese and undergone MBS. The study design of most of the included studies (49 studies) were prospective and 11 were retrospective. Included individuals were aged between 16.3 and 65 years. Also, included patients have experienced several type of MBS including Roux-en-Y GB, SG, a combination of GB and SG, and laparoscopic adjustable gastric banding (LAGB). The evaluated outcomes were as follow: TT, FT, E_2_, SHBG, PRL, and IIEF.

**Table 1:** Basic characteristics of the included studies.

Study	Year	Study design	Country	Sample size	Age	Surgery type	Follow-up (months)	Assay method	Outcome indicators
Gao et al.	2024	Prospective	China	34	25.59	SG	3, 6, 12	Immunoassay	IIEF-5
Bombardieri et al.	2024	Retrospective	Italy	184	45.5 ± 11.9	GB + SG	6	Immunoassay	TT
Javani et al.	2023	Prospective	Iran	20	34.74	GB + SG	6	Immunoassay	TT, IIEF-5
Kaur et al.	2023	Prospective	America	6	18.21 ± 0.84	SG	2 y	—	TT, E_2_, SHBG
Sultan et al.	2023	Prospective	Malaysia	13	30–45	Others	3, 6	—	IIEF-5
Abouelgreed et al.	2023	Prospective	United Arab Emirates	54	46 ± 4.83	SG	18	Immunoassay	TT, FT, E_2,_ SHBG, PRL
Jedamzik et al.	2023	Retrospective	Austria	170	43.2 ± 12.9	GB	3, 6, 24	—	TT, E_2,_ SHBG
Nosrati et al.	2023	Retrospective	Iran	41	39.43 ± 9.26	GB + SG	12	—	IIEF
Taskin et al.	2022	Prospective	Türkiye	166	51.5 ± 9.3	SG	6	—	TT
Chen et al.	2022	Prospective	China	59	32.1 ± 6.7	GB + SG	23.2	—	TT, E_2,_ PRL, IIEF
Farup et al.	2022	Prospective	Norway	13	47.7 ± 8.5	GB + SG	12	—	TT, SHBG
Miñambres et al.	2022	Prospective	Spain	12	45 ± 4.87	GB + SG	6, 18	Immunoassay	TT, FT, E_2,_ SHBG, PRL
Dhindsa et al.	2022	Prospective	United States	34	17.4 ± 1.5	GB + SG	6, 60	—	TT, FT, E_2,_ SHBG
Dilimulati et al.	2021	Retrospective	China	55	28.9 ± 7.49	SG	3, 6, 12	Immunoassay	TT
Fahmy et al.	2021	Prospective	Egypt	82	39 ± 14.6	SG	12	Immunoassay	IIEF
Fariello et al.	2021	Prospective	Brazil	15	20–50	GB	3, 9, 12	—	TT, FT, E_2,_ SHBG
Gokalp et al.	2021	Prospective	Türkiye	31	34	SG	12	Radioimmunoassay	TT, E_2,_ IIEF
Machado et al.	2021	Prospective	Brazil	33	36.3 ± 8.1	GB + SG	6	Immunoassay	TT, FT, E_2,_ SHBG, PRL
Öncel et al.	2021	Prospective	Türkiye	40	35.70 ± 4.22	SG	6	—	IIEF-5
Velde et al.	2021	Prospective	Belgium	14	52	GB	1.5, 6, 12	Immunoassay	TT, FT, E_2,_ SHBG
Sarhan et al.	2021	Prospective	Egypt	48	38.56 ± 10.8	GB + SG	12	Immunoassay	TT, IIEF
Vincenzo et al.	2020	Prospective	Italy	29	40.5 ± 9.9	SG	1	—	TT, E_2_
Karaca et al.	2020	Prospective	Türkiye	36	51.5 ± 6.39	SG	6, 12	Immunoassay	IIEF
Arolfo et al.	2020	Prospective	Italy	44	43.45	GB + SG	12	—	TT, FT, E_2,_ SHBG, PRL
Beiglböck et al.	2020	Retrospective	Austria	49	42.1 ± 11.93	GB + SG	18	Immunoassay	TT, E_2,_ SHBG
Calderón et al.	2020	Prospective	Dominican	20	40 ± 8	GB + SG	24	Radioimmunoassay	TT, FT, E_2,_ SHBG
Cobeta et al.	2020	Prospective	Spain	40	48.5 ± 9.24	GB + SG	6	Immunoassay	TT, FT
Celikcan et al.	2019	Prospective	Türkiye	10	35.4 ± 5.4	LAGB	6	—	IIEF-5
Carette et al.	2019	Prospective	France	46	38.9 ± 7.9	GB + SG	6, 12	Immunoassay	TT, E_2,_ SHBG, IIEF-5
Zhu et al.	2019	Prospective	China	56	30.8 ± 7.8	SG	12	—	TT, SHBG
Rigon et al.	2019	Retrospective	Brazil	29	42.79 ± 9.50	GB + SG	6	Immunoassay	TT, FT, SHBG
Samavat et al.	2018	Prospective	Italy	23	38 ± 9	GB	6	Immunoassay	TT, FT, E_2,_ SHBG
Chin et al.	2018	Prospective	United States	16	16.3 ± 1.2	LAGB	24	Immunoassay	TT
Liu et al.	2018	Retrospective	China	45	43.73 ± 10.13	GB	6, 12	—	TT, FT, E_2,_ SHBG
Gao et al.	2018	Prospective	China	30	33.0 ± 9.5	SG	6	—	TT, E_2,_ SHBG
Aleid et al.	2017	Prospective	British	30	46.98 ± 7.25	GB + SG	3, 6	—	IIEF
Groutz et al.	2017	Prospective	Israel	39	40.7 ± 12.4	SG	3	—	IIEF
Bardisi et al.	2016	Prospective	Qatar	46	36.65 ± 11.48	SG	12	Immunoassay	TT, E_2,_ PRL
Boonchaya et al.	2016	Prospective	Thailand	29	30.8 ± 8.1	GB + SG	1, 6	Immunoassay	TT, FT, E_2,_ SHBG
Legro et al.	2015	Prospective	United States	6	36.22 ± 3.9	GB	3, 6, 12	Immunoassay	TT, E_2,_ SHBG
Bekaert et al.	2015	Prospective	Belgium	14	51 ± 12	GB	24	—	TT, FT, E_2_, SHBG
Sarwer et al.	2015	Prospective	United States	32	46.84 ± 10.66	GB	48	—	TT, SHBG, IIEF
Samavat et al. (A)	2014	Prospective	Italy	55	42.3 ± 11.6	GB + SG	6, 12	Immunoassay	TT, FT, E_2,_ SHBG
Samavat et al. (B)	2014	Prospective	Italy	76	42 ± 11	GB	9	Immunoassay	TT, FT, E_2,_ SHBG
Aarts et al.	2014	Prospective	Netherland	24	43.32 ± 1.76	GB	12	—	TT, FT, E_2_, SHBG
Calderón et al.	2014	Prospective	Spain	35	39.29 ± 9.42	GB + SG	6	Radioimmunoassay	TT, FT, E_2,_ SHBG
Li et al.	2014	Retrospective	China	39	45.2	GB	12	—	IIEF-5
Mihalca et al.	2014	Prospective	Romania	28	43.07 ± 9.56	SG	6	—	TT, SHBG
Facchiano et al.	2013	Prospective	Italy	20	37.95	GB	6	Immunoassay	TT, FT, E_2_, SHBG
Ippersiel et al.	2013	Prospective	Belgium	21	43	GB + SG	3, 12	—	TT, FT, E_2,_ SHBG
Luconi et al.	2013	Prospective	Italy	24	39.96 ± 12.21	GB	6, 12	Immunoassay	TT, E_2,_ SHBG
Carretero et al.	2013	Prospective	Spain	20	40 ± 10.34	GB	6	—	TT, FT, E_2,_ SHBG
Mora et al.	2013	Prospective	Spain	39	43.5	GB + SG	12	Radioimmunoassay	TT, FT, E_2,_ SHBG, PRL
Pellitero et al.	2012	Prospective	Spain	33	40.5 ± 9.9	GB + SG	12	Immunoassay	TT, FT, E_2,_ SHBG, PRL
Woodard et al.	2012	Prospective	America	64	48.1 ± 1.3	GB	12	—	TT
Ranasinghe et al.	2011	Retrospective	Australia	34	52.8 ± 9.33	GB	31.79	—	IIEF
Hammoud et al.	2009	Prospective	United States	22	48.9 ± 1.2	GB	24	Immunoassay	TT, FT, E_2_
Alagna et al.	2006	Prospective	Italy	20	21–63	Others	12	—	TT, E_2_
Bastounis et al.	1998	Prospective	Greece	19	34.7 ± 7.7	Others	12	—	TT, FT, E_2,_ SHBG

### 3.2 MBS and TT and FT

The included patients were followed in 3-time point including: 1–3, 6–9, and ≥12 months following MBS. Eight studies have reported the TT levels at 1–3 month, 22 at 6–9 month, and 29 at and ≥12 months pre and post MBS.

**Figure 2:**
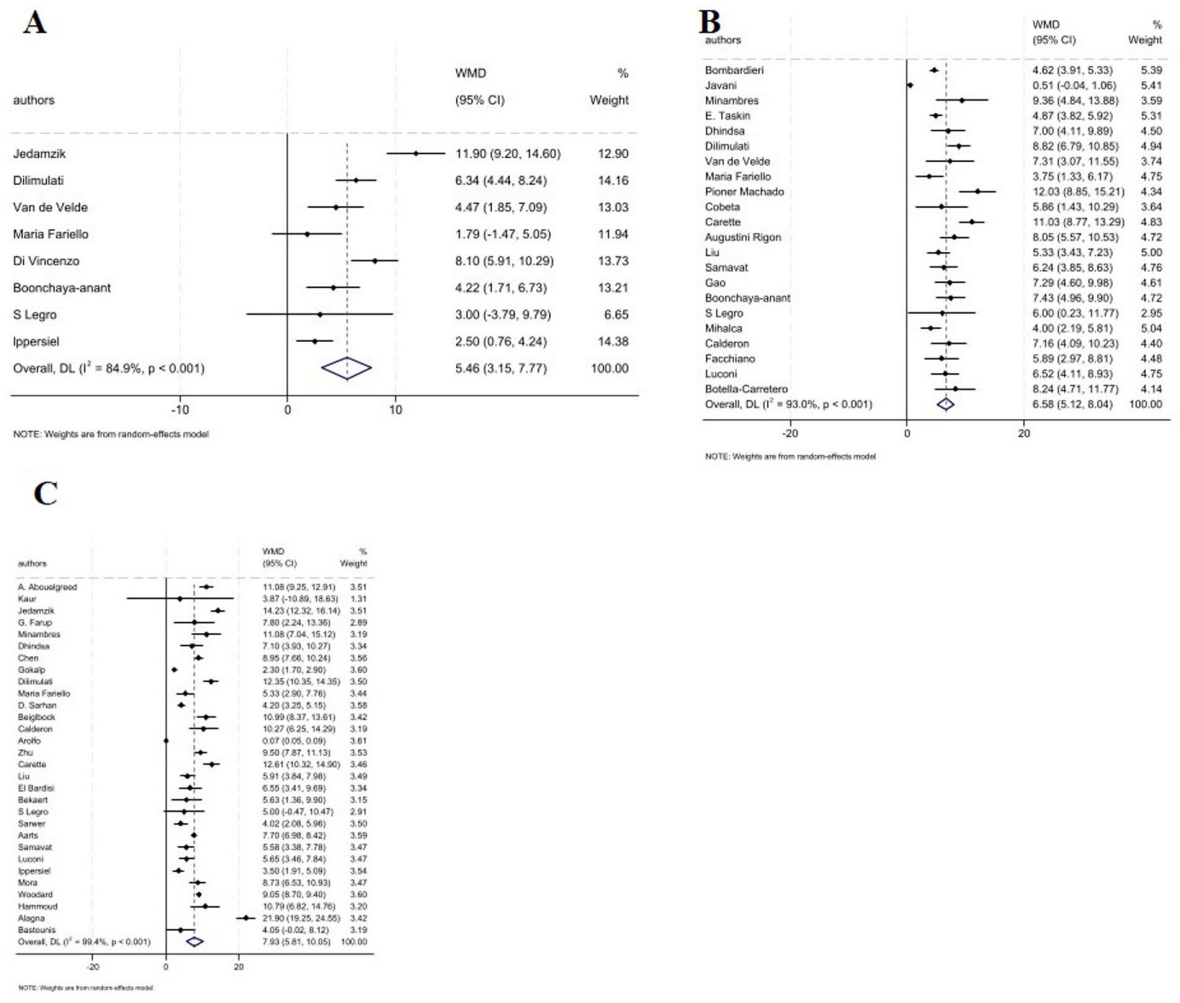
Forest plots for the effect of bariatric surgery on TT at **(A)** 1–3 M, **(B)** 6–9 M, **(C)** ≥ 12 M.

Analysis revealed a significant increase in serum TT levels following MBS across all post-operative intervals: 1–3 months (WMD: 5.46 nmol/L, 95% CI: 3.15, 7.77; *p* < 0.001); 6–9 months (WMD: 6.58 nmol/L, 95% CI: 5.12, 8.04, *p* < 0.001); and ≥12 months (WMD: 8.02 nmol/L, 95% CI: 5.85, 10.18, *p* < 0.001) (Figures 2A–C). However, subgroup analysis based on type of the surgery showed significant increase in serum TT levels in GB, SG, and GB plus SG across all time points (Table 2). Notably, at 6–9 months, GB-only studies demonstrated highly consistent effects (WMD: 5.82 nmol/L; 95% CI: 4.85–6.78; *I*^2^ = 0.0%) (Table 2).

**Table 2:** Subgroup analysis based on type of surgery.

Subgroup	No. of studies	WMD (95% CI)	*p*-value	Meta-analysis results	
				*I*^2^ (%)	*p*-value
Sex hormones					
TT (nmol/L) at 1–3 months					
*Type of surgery*					
GB	4	5.50 (0.46, 10.54)	0.032	88.7	<0.001
SG	2	7.13 (5.42, 8.85)	<0.001	29.4	0.234
GB and SG	2	3.11 (1.50, 4.73)	<0.001	17.9	0.270
TT (nmol/L) at 6–9 months					
*Type of surgery*					
GB	8	5.82 (4.85, 6.78)	<0.001	0.0	0.564
SG	4	6.11 (4.04, 8.18)	<0.001	81.2	0.001
GB and SG	10	7.17 (4.61, 9.74)	<0.001	96.1	<0.001
TT (nmol/L) at >12 months					
*Type of surgery*					
GB	10	7.51 (6.01, 9.00)	<0.001	90.2	<0.001
SG	6	8.04 (3.36, 12.73)	0.001	97.5	<0.001
GB and SG	11	7.64 (4.50, 10.78)	<0.001	98.3	<0.001
Others	2	13.04 (−4.45, 30.54)	0.144	98.1%	<0.001
FT (pmol/L) at 1–3 months					
*Type of surgery*					
GB	2	9.26 (−21.81, 40.32)	0.559	0.0	0.576
GB and SG	2	11.98 (−15.12, 39.08)	0.386	0.0	0.401
FT (pmol/L) at 6–9 months					
*Type of surgery*					
GB	6	41.05 (21.42, 60.67)	<0.001	13.4	0.329
GB and SG	7	86.12 (56.17, 116.07)	<0.001	50.9	0.057
FT (pmol/L) at >12 months					
*Type of surgery*					
GB	7	71.53 (5.47, 137.58)	0.034	98.5	<0.001
SG	1	140. (126.66, 153.34)	<0.001	0.0	<0.001
GB and SG	7	86.62 (49.55, 123.69)	<0.001	78.6	<0.001
Others	1	7.56 (−3.66, 18.78)	0.187	0.0	<0.001
E_2_ (pmol/L) at 1–3 months					
*Type of surgery*					
GB	4	3.83 (−5.23, 12.88)	0.407	44.5	0.144
SG	1	−28.30 (−49.33, −7.27)	0.008	0.0	<0.001
GB and SG	2	1.07 (−16.66, 18.80)	0.906	0.0	0.892
E_2_ (pmol/L) at 6–9 months					
*Type of surgery*					
GB	9	−14.44 (−25.39, −3.50)	0.010	68.5	0.001
SG	1	−32.67 (−132.92, 67.58)	0.523	0.0	<0.001
GB and SG	6	−20.16 (−49.86, 9.55)	0.184	83.1	<0.001
E_2_ (pmol/L) at >12 months					
*Type of surgery*					
GB	9	−12.06 (−16.24, −7.88)	<0.001	39.8	0.102
SG	4	−5.23 (−7.71, −2.75)	<0.001	0.0	0.993
GB and SG	10	−9.89 (−18.19, −1.58)	0.020	28.0	0.187
Others	2	−89.34 (−123.41, −55.27)	<0.001	0.0	0.529
SHBG (nmol/L) at 1–3 months					
*Type of surgery*					
GB	4	16.17 (6.82, 25.53)	0.001	67.6	0.026
GB and SG	2	11.87 (−0.41, 24.14)	0.058	77.5	0.035
SHBG (nmol/L) at 6–9 months					
*Type of surgery*					
GB	9	19.82 (14.11, 25.53)	<0.001	68.6	0.001
SG	2	10.83 (3.85, 17.81)	0.002	77.1	0.037
GB and SG	7	18.67 (8.04, 29.29)	0.001	94.7	<0.001
SHBG (nmol/L) at >12 months					
*Type of surgery*					
GB	9	25.65 (14.29, 37.01)	<0.001	95.6	<0.001
SG	3	16.16 (11.92, 20.39)	<0.001	50.5	0.133
GB and SG	10	18.92 (9.82, 28.01)	<0.001	95.5	<0.001
Others	1	23.64 (10.63, 36.65)	<0.001	0.0	<0.001

WMD, weighted mean difference; CI, confidence interval.

**Figure 3:**
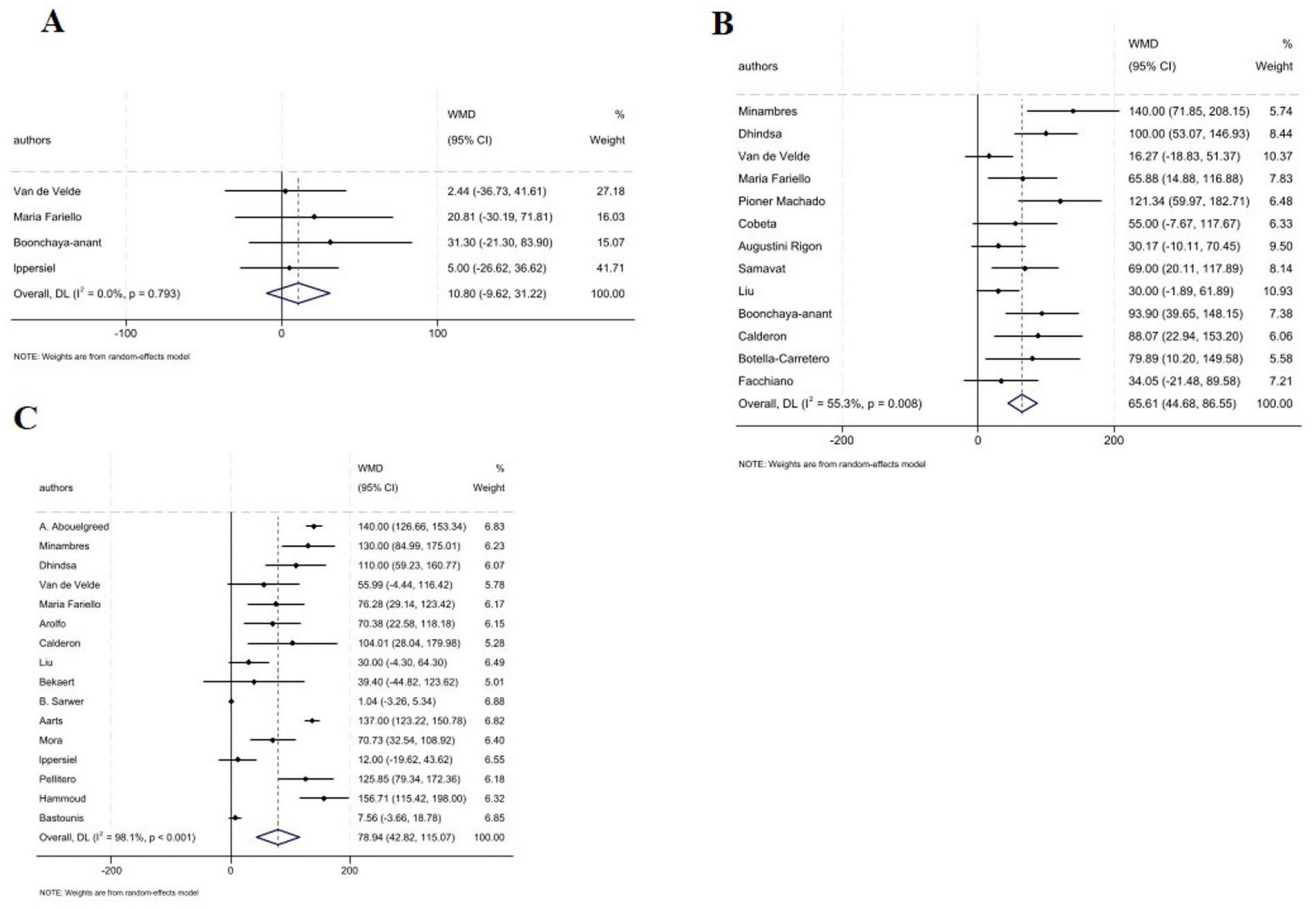
**(A–C)** Forest plots for the effect of bariatric surgery on FT at **(A)** 1–3 M, **(B)** 6–9 M, **(C)** ≥ 12 M.

While, MBS was associated with non-significant effect in FT at 1–3 months (4 studies, WMD: 10.8 nmol/L, 95% CI: −9.62, 31.22, *p* = 0.300), and significant increase at 6–9 months (13 studies, WMD: 66.61 nmol/L, 95% CI: 44.68, 86.55, *p* < 0.001), and ≥12 months (16 studies, WMD: 78.94 nmol/L, 95% CI: 42.82, 115.07, *p* < 0.001) (Figures 3A–C). In addition, subgroup analysis based on type of surgery revealed that serum FT levels has not been affected following surgery in GB and GB plus SG type at 1–3 months. While, it was increased in all type of surgery at 6–9, and ≥12 months (Table 2). FT effect estimates stratified by surgery type and follow-up duration presented significant increases emerging at 6–9 months across all procedure types including (GB; WMD: 41.05 nmol/L;, 95% CI: 21.42, 60.67, *p* < 0.001) and (GB + SG; WMD: 86.12 nmol/L;, 95% CI: 56.17, 116.07, *p* < 0.001) and persisting at ≥12 months (GB + SG; WMD: 86.62 nmol/L;, 95% CI: 49.55, 123.69, *p* < 0.001).

### 3.3 MBS and E_2_

**Figure 4:**
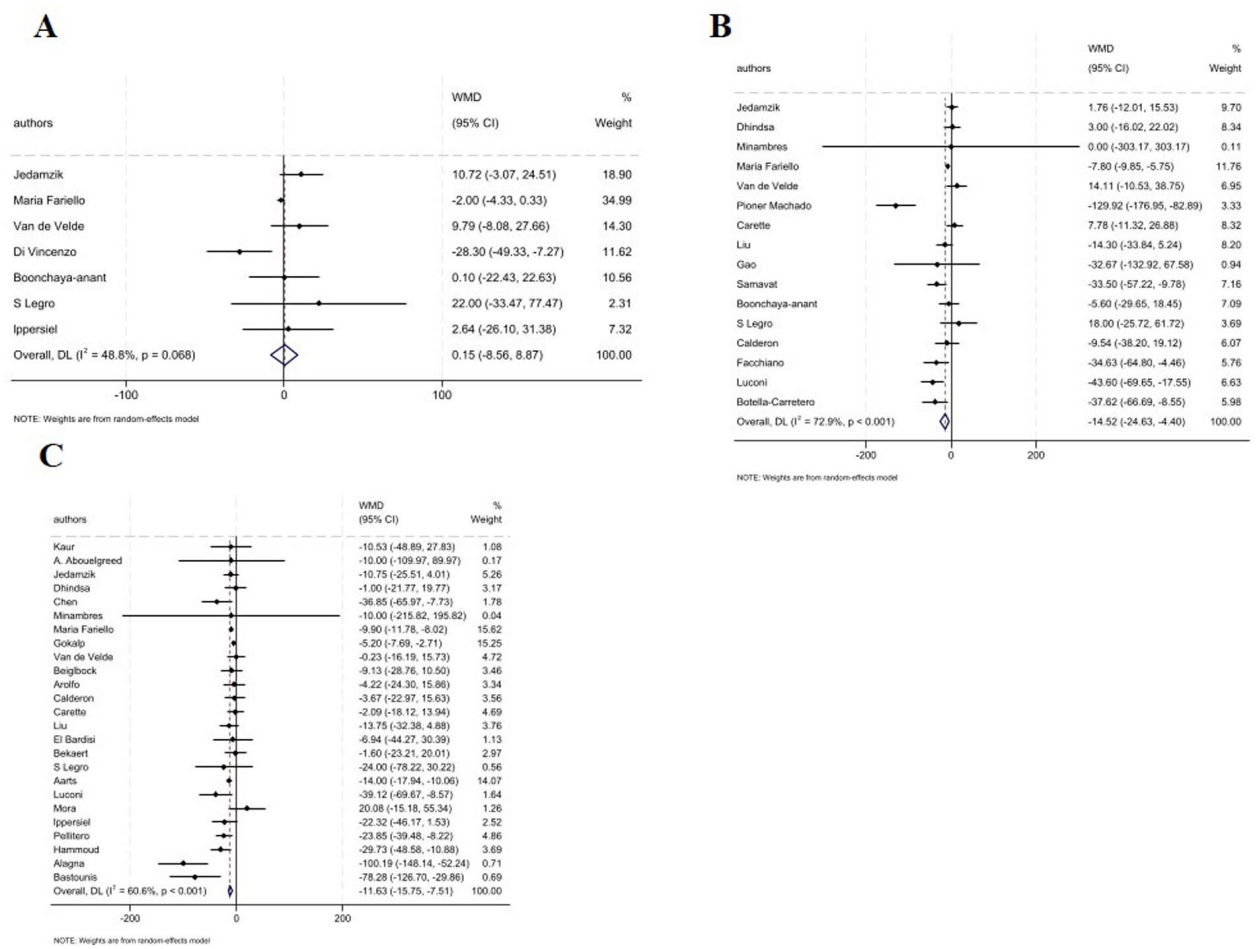
Forest plots for the effect of bariatric surgery on E_2_ at **(A–C)**: **(A)** 1–3 M, **(B)** 6–9 M, **(C)** ≥ 12 M.

The pooled analysis indicated that the serum E_2_ levels was not affected following MBS at 1–3 months (7 studies; WMD: 0.15 pmol/L, 95% CI: −8.56, 8.87, *p* = 0.973). In contrast, analysis revealed a significant decrease in E_2_ levels at 6–9 months (16 studies; WMD: −14.52 nmol/L, 95% CI: −24.63, −4.40, *p* = 0.005), and ≥12 months (25 studies; WMD: −11.63 nmol/L; 95% CI: −15.75, −7.51, *p* < 0.001) (Figures 4A–C). Moreover, subgroup analysis indicated that the type of surgery whether GB or SG did not significantly influence the overall effect on E_2_ levels at 1–3 months. Whereas, based on subgroup analysis GB type showed significant decline in E_2_ levels at 6–9 months (WMD: −14.44 nmol/L; 95% CI: −25.39, −3.50, *p* < 0.001), nut no effect in GB plus SG type. Importantly, at ≥12 months all surgery types were accompanied with significant reduction in E_2_ levels (Table 2).

### 3.4 MBS and SHBG

**Figure 5:**
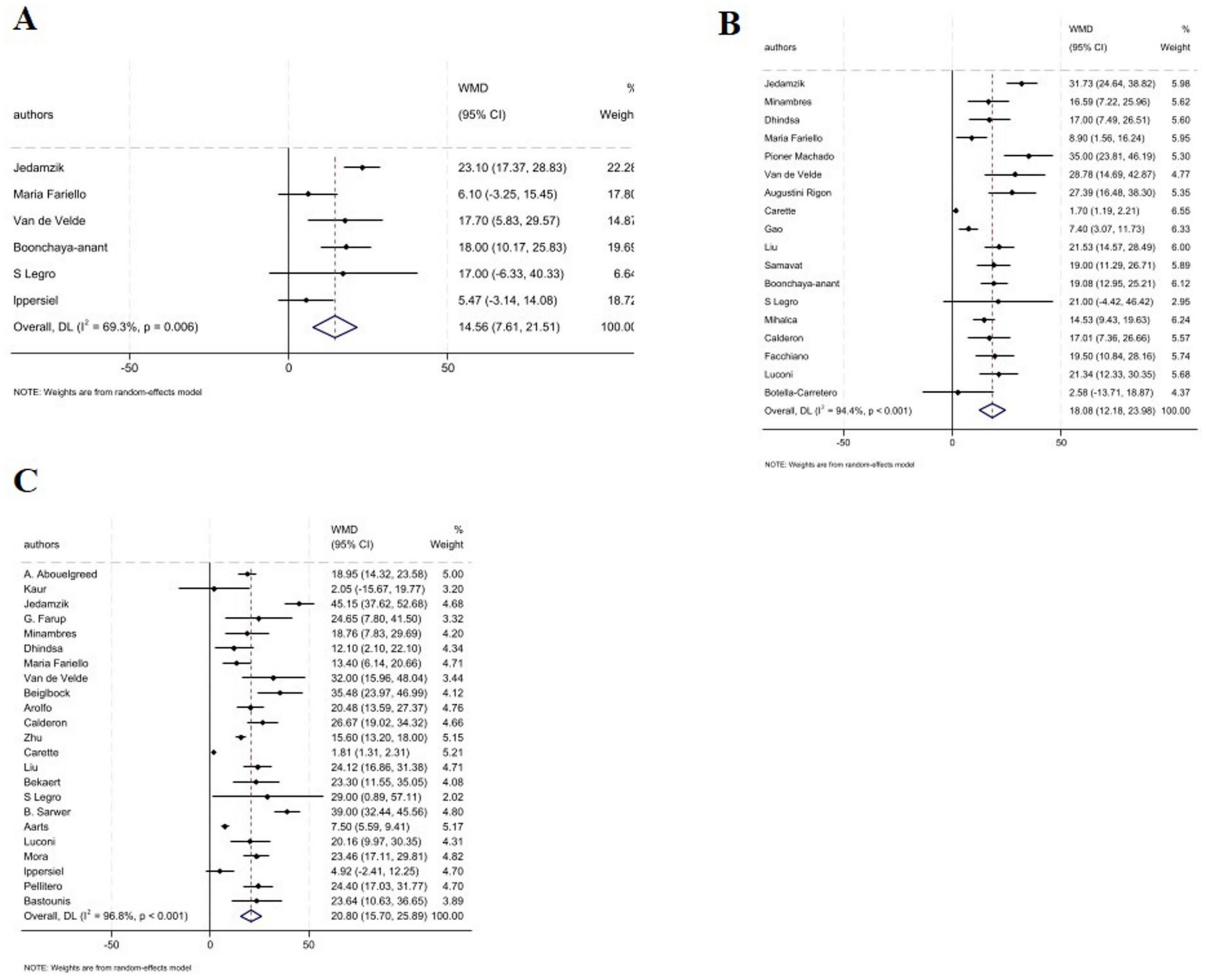
Forest plots for the effect of bariatric surgery on SHBG at **(A)** 1–3 M, **(B)** 6–9 M, **(C)** ≥ 12 M.

The combined effect of included studies for SHBG value showed elevated levels of SHBG following bariatric surgery across all time points: 1–3 months (6 studies; WMD: 14.56 nmol/L, 95% CI: 7.61, 21.51; *p* < 0.001); 6–9 months (18 studies; WMD: 18.08 nmol/L, 95% CI: 12.18, 23.98, *p* < 0.001); and ≥12 months (23 studies; WMD: 23.64 nmol/L, 95% CI: 10.63, 36.65, *p* < 0.001) (Figures 5A–C). In addition, subgroup analysis indicated that any type of surgery is accompanied with a significant increase in the SHBG levels across all post-operative intervals except for GB plus SG surgery at 1–3 months which failed to show any significant changes in SHBG levels (Table 2).

### 3.5 MBS and PRL

**Figure 6:**
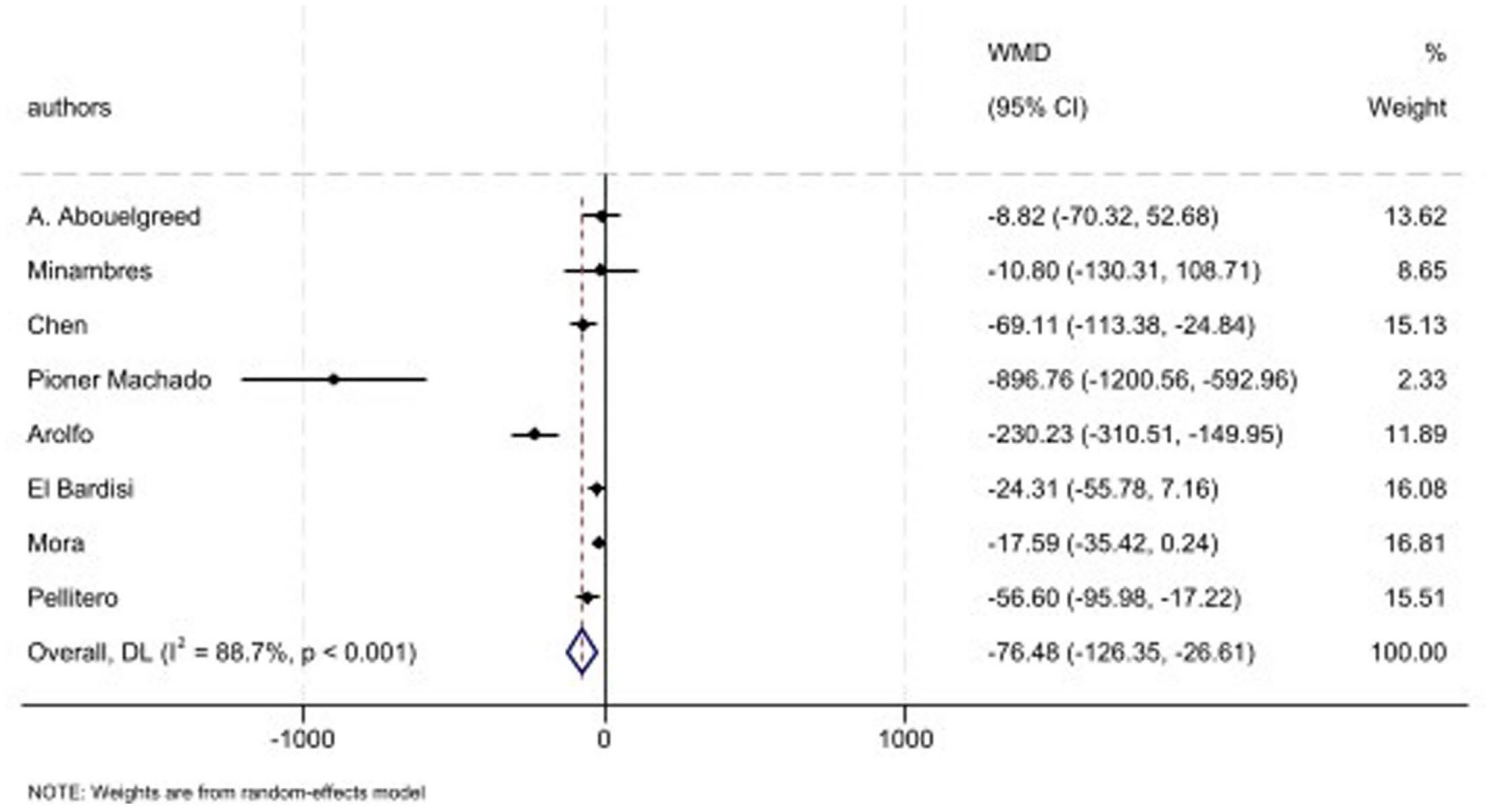
Forest plots for the effect of bariatric surgery on PRL.

Eight studies have evaluated the PRL levels and pooled analysis demonstrated that PRL levels was decreased (WMD: −76.48 mIU/L, 95% CI: −126.35, −40.0, *p* = 0.003; *I*^2^: 88.7%, *p* < 0.001) (Figure 6). Also, subgroup analysis based on type of surgery showed similar results in each surgery types including SG, and GB plus SG (Table 2).

### 3.6 MBS and IIEF score

**Figure 7:**
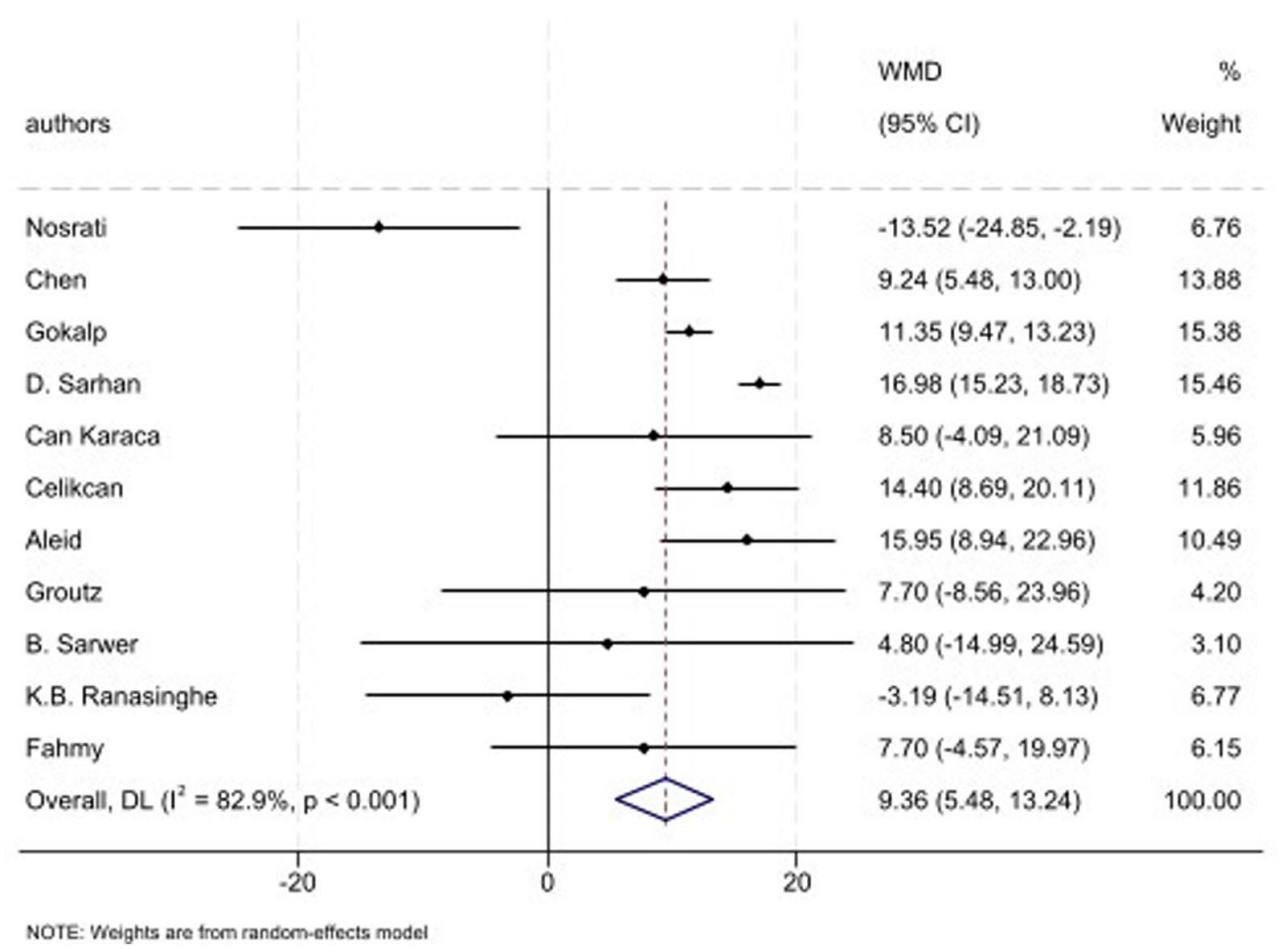
Forest plots for the effect of bariatric surgery on IIEF.

Eleven studies have evaluated the semen parameters and reported the IIEF levels. The combined effect of included studies showed that the IIEF score is increased following surgery (WMD: 9.36, 95% CI: 5.48, 13.24, *p* < 0.001; *I*^2^: 82.9%, *p* < 0.001) (Figure 7). Furthermore, subgroup analysis showed that SG, GB plus SG, and LAGB have shown increased level of IIEF score in post-operative state too. While, GB could not reveal significant changes following MBS (Table 2).

### 3.7 Sensitivity analysis and publication bias

This study employed sensitivity analysis to present robust findings. It has been demonstrated that leave-one-out approach did not show any study to affect the overall results which is a proof of the results robustness.

The publication bias was evaluated using Begg’s test and it has been shown that Begg’s test was >0.05 for all study outcomes across all post-operative intervals except for TT at >12 months (*p* = 0.002), FT at 6–9 months (*p* = 0.044), and SHBG at >12 months (*p* = 0.042). In addition, funnel plots have been provided for visual inspection too (Supplementary Figure 1).

### 3.8 Quality evaluation, meta-regression, and GRADE assessment

The results of quality assessment of included studies using NOS is provided in Table 3. Most of the included studies were scored ≥6 which offers an acceptable quality for studies. Meta-regression analyses revealed that none of the examined covariates, including surgical type, follow-up duration, baseline BMI, or study quality, significantly explained the observed heterogeneity (*p* > 0.05 for all).

**Table 3:** The quality assessment of the included studies using NOS.

Study	Year	Selection				Comparability	Outcome			Overall score
		#1	#2	#3	#4	#1	#1	#2	#3	
Gao et al.	2024	*	*	*	—	*	*	—	—	5
Bombardieri et al.	2024	*	*	*	—	*	*	*	—	6
Javani et al.	2023	*	*	*	—	*	*	*	—	6
Kaur et al.	2023	*	*	*	—	*	*	—	*	6
Sultan et al.	2023	*	*	*	—	*	*	*	—	6
Abouelgreed et al.	2023	*	*	*	—	*	*	—	*	6
Jedamzik et al.	2023	*	*	*	—	*	*	*	—	6
Nosrati et al.	2023	*	*	*	—	*	*	—	*	6
Taskin et al.	2022	*	*	*	—	*	*	*	—	6
Chen et al.	2022	*	*	*	—	*	*	*	*	7
Farup et al.	2022	*	*	*	—	*	*	—	*	6
Miñambres et al.	2022	*	*	*	—	*	*	*	—	6
Dhindsa et al.	2022	*	*	*	—	*	*	*	—	6
Dilimulati et al.	2021	*	*	*	—	*	*	*	—	6
Fahmy et al.	2021	*	*	*	—	*	*	—	*	6
Fariello et al.	2021	*	*	*	—	*	*	*	*	7
Gokalp et al.	2021	*	*	*	—	*	*	*	*	7
Velotti et al.	2021	*	*	*	—	*	*	*	*	7
Machado et al.	2021	*	*	*	—	*	*	*	*	7
Öncel et al.	2021	*	*	*	—	*	*	*	*	7
Velde et al.	2021	*	*	*	*	*	*	*	*	8
Sarhan et al.	2021	*	*	*	—	*	*	*	*	7
Vincenzo et al.	2020	*	*	*	*	*	*	—	—	6
Karaca et al.	2020	*	*	*	—	*	*	—	—	5
Arolfo et al.	2020	*	*	*	—	*	*	*	*	7
Beiglböck et al.	2020	*	*	*	—	*	*	*	*	7
Calderón et al.	2020	*	*	*	—	*	*	*	—	6
Cobeta et al.	2020	*	*	*	—	*	*	*	*	7
Celikcan et al.	2019	*	*	*	*	*	*	—	—	6
Carette et al.	2019	*	*	*	—	*	*	*	—	6
Zhu et al.	2019	*	*	*	*	*	*	*	*	8
Rigon et al.	2019	*	*	*	*	*	*	*	—	7
Samavat et al.	2018	*	*	*	—	*	*	*	—	6
Chin et al.	2018	*	*	*	—	*	*	*	—	6
Liu et al.	2018	*	*	*	—	*	*	*	—	6
Gao et al.	2018	*	*	*	—	*	*	*	*	7
Aleid et al.	2017	*	*	*	—	*	*	*	—	6
Groutz et al.	2017	*	*	*	—	*	*	—	*	6
Bardisi et al.	2016	*	*	*	—	*	*	*	—	6
Boonchaya et al.	2016	*	*	*	—	*	*	*	—	6
Legro et al.	2015	*	*	*	—	*	*	*	—	6
Bekaert et al.	2015	*	*	*	—	*	*	—	*	6
Sarwer et al.	2015	*	*	*	—	*	*	*	*	7
Samavat et al.(A)	2014	*	*	*	—	*	*	*	*	7
Samavat et al.(B)	2014	*	*	*	—	*	*	*	—	6
Aarts et al.	2014	*	*	*	—	*	*	—	*	6
Calderón et al.	2014	*	*	*	—	*	*	*	*	7
Li et al.	2014	*	*	*	—	*	*	*	—	6
Mihalca et al.	2014	*	*	*	—	*	*	—	—	5
Facchiano et al.	2013	*	*	*	*	*	*	*	—	7
Ippersiel et al.	2013	*	*	*	—	*	*	*	*	7
Luconi et al.	2013	*	*	*	—	*	*	*	—	6
Carretero et al.	2013	*	*	*	—	*	*	*	—	6
Mora et al.	2013	*	*	*	—	*	*	*	*	7
Pellitero et al.	2012	*	*	*	—	*	*	—	*	6
Woodard et al.	2012	*	*	*	—	*	*	*	*	7
Ranasinghe et al.	2011	*	*	*	—	*	*	—	*	6
Hammoud et al.	2009	*	*	*	—	*	*	—	*	6
Alagna et al.	2006	*	*	*	—	*	*	*	*	7
Bastounis et al.	1998	*	*	*	—	*	*	*	*	7

*Mean one points.

The quality of evidence has been assessed using GRADE approach (Table 4). Accordingly, E_2_ at >12 M rated as high quality of evidence. However, other study outcomes were considered as moderate or low.

**Table 4:** Summary of findings and quality of evidence assessment using the GRADE approach.

	No of studies	ES (95% CI)	Risk of bias	Inconsistency	Indirectness	Imprecision	Publication bias	Quality of evidence
TT at 1–3 M	7	5.46 (3.15, 7.77)	Not serious	Serious	Not serious	Not serious	Not serious	Moderate
TT at 6–9 M	22	6.58 (5.12, 8.04)	Not serious	Serious	Not serious	Not serious	Not serious	Moderate
TT at >12 M	29	8.02 (5.85, 10.18)	Not serious	Serious	Not serious	Not serious	Serious	Low
FT at 1–3 M	4	10.80 (−9.62, 31.22)	Not serious	Not serious	Not serious	Serious	Not serious	Moderate
FT at 6–9 M	13	65.61 (44.68, 86.55)	Not serious	Not serious	Not serious	Not serious	Serious	Moderate
FT at >12 M	16	78.94 (42.82, 115.07)	Not serious	Serious	Not serious	Not serious	Not serious	Moderate
E_2_ at 1–3 M	7	0.15 (−8.56, 8.87)	Not serious	Not serious	Not serious	Serious	Not serious	Moderate
E_2_ at 6–9 M	16	−14.52 (−24.63, −4.40)	Not serious	Serious	Not serious	Not serious	Not serious	Moderate
E_2_ at >12 M	25	−11.63 (−15.75, −7.51)	Not serious	Not serious	Not serious	Not serious	Not serious	High
SHBG at 1–3 M	6	14.56 (7.61, 21.51)	Not serious	Serious	Not serious	Not serious	Not serious	Moderate
SHBG at 6–9 M	18	18.08 (12.18, 23.98)	Not serious	Serious	Not serious	Not serious	Not serious	Moderate
SHBG at >12 M	23	20.80 (15.70, 25.89)	Not serious	Serious	Not serious	Not serious	Serious	Low
PRL	8	−76.48 (−126.35, −26.61)	Not serious	Serious	Not serious	Not serious	Not serious	Moderate
IIEF	11	9.36 (5.48, 13.24)	Not serious	Serious	Not serious	Not serious	Not serious	Moderate

## 4 Discussion

The adverse effects of obesity on male reproductive function represent a significant and growing global public health concern. MBS has garnered increasing attention for its potential as an effective therapeutic strategy for obesity. In addition, evidence indicates that MBS have beneficial effects on reproductive health outcomes too (16). Accordingly, this meta-analysis synthesizes the available evidence to evaluate the impact of MBS on male sex hormone profiles, semen parameters, and sexual function. Our findings illustrated that MBS leads to significant improvements in the sex hormone status among obese men. However, its effect on semen quality appears to be challenging. The MBS has distinct effects on semen quality based on various surgical techniques and follow-up duration.

Our findings demonstrated that obese men have increased levels of TT and FT across all post-operative intervals. This consistent elevation in circulating androgens offers endocrinological responses to MBS. Obese individuals experience hypogonadotropic hypogonadism which is characterized by reduced testosterone and testicular function (15, 17, 18). Likewise, the MBS is able to reverse this process and exert its beneficial effects on TT and FT levels. Also, mechanistically MBS contribute to reduced adipose tissue which is accompanied with diminished activity of aromatase to converts androgens to estrogens (19, 20).

Despite the consistency in the direction and statistical significance of MBS, there was a statistical significance heterogeneity in pooled estimates for TT and FT. Subgroup analysis at 6–9 months, based on surgical procedure revealed that GB-type surgery was associated with very low heterogeneity (*I*^2^ = 0.0%) and a significant effect. This finding suggests that a standardized surgical approach, such as GB-type procedures, may lead to a more consistent hormonal response. For FT, heterogeneity measurement is critical too. It has been shown that, at 1–3 months, both GB- and (GB + SG)-type surgery presented low heterogeneity (*I*^2^ = 0.0%) with non-significant effects. It means that, no heterogeneity could not affect the FT levels in postoperative period and it may need more time to be altered. Furthermore, it was demonstrated that FT levels were significantly affected at 6–9 months and >12 months following GB-type, (GB + SG)-type, and SG-type surgeries, with low heterogeneity observed for each (*I*^2^ = 0.0%).

A decrease in E_2_ levels is ultimately associated with an increase in testosterone synthesis (21). An increase in testosterone of 8.02 nmol/L (over ≥12 months) is clinically significant, as it is sufficient to move a patient from the hypogonadal range (<8 nmol/L) into the eugonadal range (>12 nmol/L). For erectile function, the 9.36-point improvement in IIEF-5 score substantially exceeds the minimal clinically important difference (MCID) of 4-5 points, indicating a tangible and meaningful improvement for the patient. The 11.63 pmol/L reduction in E_2_ represents a normalization of the hyperestrogenic state typical of male obesity, thereby relieving inhibitory feedback on the HPG axis (22). The 23.64 nmol/L increase in SHBG reflects a resolution of underlying metabolic dysfunction and the normalization of hepatic protein synthesis (23).

Moreover, this surgical intervention was associated with decreased E_2_ levels in obese men at 6–9 months and beyond 12 months of follow-up. This finding constitutes a complementary finding to the abovementioned androgens. Increased circulating estrogens in obesity suppresses LH secretion and strengthens testosterone deficiency (24). Therefore, the decrease in E_2_ after surgery follows the elimination of the inhibitory effect of this signal. In this context, several underlying mechanisms are probable. It has been proposed that reduced availability of aromatase substrates, downregulation of aromatase activity, and altered SHBG may be involved in the reduced level of E_2_ (25–27). Increased levels of TT, FT, and decreased levels of E_2_ simultaneously suggest hypothalamic-pituitary-gonadal (HPG) axis homeostasis (28). Moreover, based on subgroup analysis, all subgroups demonstrated significant E_2_ reductions with low heterogeneity (GB: *I*^2^ = 39.8%; SG: *I*^2^ = 0.0%; GB + SG: *I*^2^ = 28.0%). Over time, heterogeneity decreased and the overall effect became statistically significant across all surgery types.

It is worth noting that SHBG levels are increased in post-operative state in obese men across all time points which is indicating a hallmark endocrinological improvement (29, 30). This finding shares a valuable effect of surgery in obese men. The postoperative increase in SHBG is attributable to several probable mechanisms. Previous studies have introduced the insulin resistance as a characteristic of obesity (31, 32). Whereas, significant reduction in insulin concentrations happens in postoperative state and contribute to improved insulin sensitivity (33, 34). However, the role of improved inflammatory cytokine could not be ignored in this regard (35, 36). Also, it seems that SHBG increase is integrated to observed increase in TT and FT levels androgen recovery, contributing to TT elevation.

The postoperative decline in PRL concentrations signifies the improvement of metabolic disturbance in obese patients. The role of PRL in male reproductive health explain the observed effect (37). The PRL modulates gonadotropin secretion in men (38). Accordingly, MBS is able to restore LH pulsatility and testosterone secretion in male (39, 40).

The recent meta-analysis by Puia et al. (41) shared distinct results in term of TT and SHBG levels in men. In contrast, our study employed a leave-one-out sensitivity analysis, which confirmed that no single study disproportionately influenced the pooled estimates, indicating robust and stable results. Additionally, we applied the GRADE framework to assess certainty of evidence; the findings were downgraded to moderate quality, reflecting methodological rigor and transparency. The discrepancy between our findings and those of Puia et al. may be attributed to several key differences: (i) the smaller number of studies included in the prior meta-analysis, which may have limited statistical power and precision; (ii) heterogeneity in study populations (e.g., baseline testosterone status, age, comorbidities); (iii) variations in surgical techniques; (iv) differences in follow-up duration, as longer-term testosterone changes may not be captured in studies with shorter observation periods; (v) study design; (vi) bile acid and microbiota signalling are affected in malaborptive procedure. Bile acids may suppress gonadotropin secretion, whereas microbiota exert direct spermatotoxic effects; (vii) nutritional deficiencies and malabsorption. Postoperative micronutrient deficiencies (specifically zinc, selenium, vitamin B12, and vitamin D) are associated with impaired spermatogenesis. Previous studies that did not report postoperative micronutrient status, this could explain more different results. These factors collectively underscore the importance of updated evidence synthesis with rigorous sensitivity and certainty assessments.

Accordingly, careful interpretation of reproductive outcomes following MBS must account for the potential role of micronutrient deficiencies. Patients undergoing GB-type procedures are at greater risk for such deficiencies compared to those undergoing SG. Several micronutrients are known to play critical roles in male reproductive function. Zinc is essential for spermatogenesis and testosterone synthesis (42). Selenium supports the antioxidant defense system and contributes to the maintenance of male reproductive health (43). Folate and vitamin B12 are involved in methylation processes, and deficiencies in these nutrients can lead to impaired spermatogenesis and increased sperm DNA fragmentation (44). Vitamin D deficiency has also been associated with impaired reproductive function, while vitamin E exerts protective effects through the prevention of oxidative damage (45, 46). Importantly, even with apparently adequate intake, deficiencies in key micronutrients such as copper, zinc, and vitamin D may develop over time. This observation underscores the importance of active, routine monitoring rather than passive supplementation alone. Individualized treatment approaches with follow-up at 3–6 month intervals may optimize reproductive outcomes. Therefore, periodic monitoring of predefined micronutrient status is strongly recommended in male patients following MBS.

Despite the important findings post-MBS, sustained weight loss and reproductive improvements are critical. A systematic review stated that 76% of SG patients had experienced significant weight regain at 6 year follow-up (47). Similarly, another meta-analysis declared overally, 49% weight regain for post-MBS and 42% weight regain for post-GB specifically (48). In this context, the Pham et al.’s (49) trial demonstrated that, FT and TT increment was persistent at 5 years. However, these improvements are guaranteed with sustained weight management in patients (49). While, Dhindsa et al.’s (50) study indicated that patients who had reported weight regain had a decrease in TT levels. Evidence supports that hormonal parameters do not revert to baseline when weight loss is sustained (50).

This study has some limitations too. First, high heterogeneity limits the generalizability of the findings. Second, the publication bias for TT at >12 months, FT at 6–9 months, and SHBG at >12 months affects the observed finding. Third, there was limited data in term of adverse effects such as micronutrient deficiency and their interaction with reproductive health. The influence of surgical type should not be overlooked. SG appears to exert a more consistent effect on SG than GB, possibly owing to the latter’s association with the malabsorption of key micronutrients such as zinc and selenium (51). The primary studies included in this meta-analysis provided limited and inconsistently reported data on micronutrient malabsorption. Consequently, we were unable to perform quantitative synthesis or subgroup analyses to assess the direct effect of specific nutrient deficiencies on study outcomes. Forth, lack of data on functional fertility endpoints (sperm DNA fragmentation, live birth rates). Fifth, the included studies span multiple WHO classification editions (4th, 5th, and 6th), which employ different reference values and methodologies. The pooled results may therefore be influenced by unrecognized differences in outcome classification across studies, and should be interpreted with caution.

## 5 Conclusion

The present meta-analysis provides a comprehensive and integrated synthesis of the available evidence examining the impact of MBS on male reproductive health. Our findings demonstrated that MBS is able to induce an increase on the levels of androgens including TT at all-time points and FT at 6–9, and >12 months follow-up duration. Moreover, it exerts favorable effects on E_2_ (at 6–9 and >12 months follow-up duration), PRL and IIEF score. In addition, SHBG level was affected following MBS significantly in obese men. However, there are many gaps which needs to shift from heterogeneous, underpowered retrospective studies that gather reproductive, nutritional, and metabolic signatures.

## Data Availability

The original contributions presented in the study are included in the article/Supplementary material, further inquiries can be directed to the corresponding author.

## References

[1] Finer N. Medical consequences of obesity. Medicine. (2015) 43:88–93. doi: 10.1016/j.mpmed.2014.11.003

[2] Ahmed SK Mohammed RA. Obesity: prevalence, causes, consequences, management, preventive strategies and future research directions. Metabol Open. (2025) 27:100375. doi: 10.1016/j.metop.2025.100375, 41041606 PMC12486175

[3] Mohajan D Mohajan HK. Obesity and its related diseases: a new escalating alarming in global health. J Innov Med Res. (2023) 2:12–23. doi: 10.56397/jimr/2023.03.04

[4] Schon SB Cabre HE Redman LM. The impact of obesity on reproductive health and metabolism in reproductive-age females. Fertil Steril. (2024) 122:194–203. doi: 10.1016/j.fertnstert.2024.04.036, 38704081 PMC11527540

[5] Barbouni K Jotautis V Metallinou D Diamanti A Orovou E Liepinaitienė A . When weight matters: how obesity impacts reproductive health and pregnancy-a systematic review. Curr Obes Rep. (2025) 14:37. doi: 10.1007/s13679-025-00629-9, 40238039 PMC12003489

[6] Kang Y Li P Yuan S Fu S Zhang X Zhang J . Progress in investigating the impact of obesity on male reproductive function. Biomedicines. (2025) 13:2054. doi: 10.3390/biomedicines13092054, 41007618 PMC12467420

[7] Chacon D Bernardino T Geraghty F Rodriguez AC Fiani B Chadhaury A . Bariatric surgery with Roux-en-Y gastric bypass or sleeve gastrectomy for treatment of obesity and comorbidities: current evidence and practice. Cureus. (2022) 14:e25762. doi: 10.7759/cureus.25762, 35812610 PMC9270090

[8] Cordero P Li J Oben J. Bariatric surgery as a treatment for metabolic syndrome. J R Coll Physicians Edinb. (2017) 47:364–8. doi: 10.4997/JRCPE.2017.414, 29537411

[9] Ji Y Lee H Kaura S Yip J Sun H Guan L . Effect of bariatric surgery on metabolic diseases and underlying mechanisms. Biomolecules. (2021) 11:1582. doi: 10.3390/biom11111582, 34827579 PMC8615605

[10] Di Vincenzo A Busetto L Vettor R Rossato M. Obesity, male reproductive function and bariatric surgery. Front Endocrinol. (2018) 9:769. doi: 10.3389/fendo.2018.00769, 30619096 PMC6305362

[11] Pozzi E Able CA Kohn TP Salonia A Ramasamy R. Testosterone levels increase following bariatric surgery-validation of preceding literature in a large-scale population analysis. Andrology. (2025) 13:431–8. doi: 10.1111/andr.13689, 38958350 PMC11867915

[12] El Bardisi H Majzoub A Arafa M AlMalki A Al Said S Khalafalla K . Effect of bariatric surgery on semen parameters and sex hormone concentrations: a prospective study. Reprod Biomed Online. (2016) 33:606–11. doi: 10.1016/j.rbmo.2016.08.008, 27569703

[13] Page MJ McKenzie JE Bossuyt PM Boutron I Hoffmann TC Mulrow CD . The PRISMA 2020 statement: an updated guideline for reporting systematic reviews. BMJ. (2021) 372:n71. doi: 10.1136/bmj.n71, 33782057 PMC8005924

[14] Stang A. Critical evaluation of the Newcastle-Ottawa Scale for the assessment of the quality of nonrandomized studies in meta-analyses. Eur J Epidemiol. (2010) 25:603–5. doi: 10.1007/s10654-010-9491-z, 20652370

[15] Basaria S. Male hypogonadism. Lancet. (2014) 383:1250–63. doi: 10.1016/S0140-6736(13)61126-5, 24119423

[16] Moxthe LC Sauls R Ruiz M Stern M Gonzalvo J Gray HL. Effects of bariatric surgeries on male and female fertility: a systematic review. J Reprod Infertil. (2020) 21:71–86. 32500010 PMC7253939

[17] Grossmann M. Hypogonadism and male obesity: focus on unresolved questions. Clin Endocrinol. (2018) 89:11–21. doi: 10.1111/cen.13723, 29683196

[18] Carrageta DF Oliveira PF Alves MG Monteiro MP. Obesity and male hypogonadism: tales of a vicious cycle. Obes Rev. (2019) 20:1148–58. doi: 10.1111/obr.12863, 31035310

[19] Frikke-Schmidt H O’Rourke RW Lumeng CN Sandoval D Seeley RJ. Does bariatric surgery improve adipose tissue function? Obes Rev. (2016) 17:795–809. doi: 10.1111/obr.12429, 27272117 PMC5328428

[20] Lecoutre S Rebière C Marcelin G Clément K. How does bariatric surgery remodel adipose tissue? Ann Endocrinol. (2024) 85:175–8. doi: 10.1016/j.ando.2024.05.008, 38871506

[21] Olasore HSA Oyedeji TA Olawale MO Ogundele OI Faleti JO-O. Relationship between testosterone-estradiol ratio and some anthropometric and metabolic parameters among Nigerian men. Metabol Open. (2023) 18:100249. doi: 10.1016/j.metop.2023.100249, 37396673 PMC10313505

[22] Rubinow KB. "Estrogens and body weight regulation in men". In: F Mauvais-Jarvis, editor. Sex and Gender Factors Affecting Metabolic Homeostasis, Diabetes and Obesity. Berlin: Springer (2017). p. 285–313. 10.1007/978-3-319-70178-3_14 PMC5835337 29224100

[23] Luo J Chen Q Shen T Wang X Fang W Wu X . Association of sex hormone-binding globulin with nonalcoholic fatty liver disease in Chinese adults. Nutr Metab. (2018) 15:79. doi: 10.1186/s12986-018-0313-8, 30455723 PMC6225668

[24] Kunimura Y Iwata K Ishii H Ozawa H. Chronic estradiol exposure suppresses luteinizing hormone surge without affecting kisspeptin neurons and estrogen receptor alpha in anteroventral periventricular nucleus. Biol Reprod. (2024) 110:90–101. doi: 10.1093/biolre/ioad129, 37774351

[25] Parween S Cancio MF Benito-Sanz S Camats N Velazquez M López-Siguero J . Molecular Basis of Aromatase Deficiency in a 46, XX Patient with Mutation of Arginine 550 to Tryptophan in POR: Expanding the Endocrine Phenotype in PORD. (2019) Available online at: https://www.preprints.org/manuscript/201909.0103/v1 (Accessed September 9, 2019).

[26] Patel S. Disruption of aromatase homeostasis as the cause of a multiplicity of ailments: a comprehensive review. J Steroid Biochem Mol Biol. (2017) 168:19–25. doi: 10.1016/j.jsbmb.2017.01.009, 28109841

[27] Guercio GV Saraco NI Costanzo M Marino RM Belgorosky A. "Human aromatase deficiency". In: Reference Module in Biomedical Sciences. Amsterdam: Elsevier (2018)

[28] Acevedo-Rodriguez A Kauffman A Cherrington B Borges C Roepke TA Laconi M. Emerging insights into hypothalamic-pituitary-gonadal axis regulation and interaction with stress signalling. J Neuroendocrinol. (2018) 30:e12590. doi: 10.1111/jne.12590, 29524268 PMC6129417

[29] Di Stasi V Maseroli E Rastrelli G Scavello I Cipriani S Todisco T . SHBG as a marker of NAFLD and metabolic impairments in women referred for oligomenorrhea and/or hirsutism and in women with sexual dysfunction. Front Endocrinol. (2021) 12:641446. doi: 10.3389/fendo.2021.641446, 33854482 PMC8040974

[30] Bourebaba N Ngo T Śmieszek A Bourebaba L Marycz K. Sex hormone binding globulin as a potential drug candidate for liver-related metabolic disorders treatment. Biomed Pharmacother. (2022) 153:113261. doi: 10.1016/j.biopha.2022.113261, 35738176

[31] Pérez MR Medina-Gómez G. Obesity, adipogenesis and insulin resistance. Endocrinol Nutr. (2011) 58:360–9. doi: 10.1016/j.endoen.2011.05.004 21778123

[32] Choo YN Ravi RN Subramaniyan V. Insulin resistance induced by obesity: mechanisms, metabolic implications and therapeutic approaches. Mol Biol Rep. (2026) 53:357. doi: 10.1007/s11033-026-11509-3, 41636917 PMC12872655

[33] Shantavasinkul PC Muehlbauer MJ Bain JR Ilkayeva OR Craig DM Newgard CB . Improvement in insulin resistance after gastric bypass surgery is correlated with a decline in plasma 2-hydroxybutyric acid. Surg Obes Relat Dis. (2018) 14:1126–32. doi: 10.1016/j.soard.2018.03.033, 29805089 PMC6153057

[34] Brzozowska MM Isaacs M Bliuc D Baldock PA Eisman JA White CP . Effects of bariatric surgery and dietary intervention on insulin resistance and appetite hormones over a 3 year period. Sci Rep. (2023) 13:6032. doi: 10.1038/s41598-023-33317-6, 37055514 PMC10102182

[35] de Souza Bett G Schuelter-Trevisol F do Nascimento RR Fernandes BB da Silva LE da Silva MR . Bariatric surgery reduces lipid profile and oxidative stress in patients with obesity: a prospective cohort study. J Metab Bariatr Surg. (2025) 14:32–42. doi: 10.17476/jmbs.2025.14.1.32, 40351819 PMC12059305

[36] Lautenbach A Stoll F Mann O Busch P Huber TB Kielstein H . Long-term improvement of chronic low-grade inflammation after bariatric surgery. Obes Surg. (2021) 31:2913–20. doi: 10.1007/s11695-021-05315-y, 33666873 PMC7934816

[37] Raut S Deshpande S Balasinor NH. Unveiling the role of prolactin and its receptor in male reproduction. Horm Metab Res. (2019) 51:215–9. doi: 10.1055/a-0859-1144, 30840999

[38] Hodson DJ Townsend J Tortonese DJ. Characterization of the effects of prolactin in gonadotroph target cells. Biol Reprod. (2010) 83:1046–55. doi: 10.1095/biolreprod.110.084947, 20720166

[39] Javani S Mosapour E Hoseine S Ashrafi A Farhadi E. Analysis of semen parameters, and hormonal changes of FSH, LH, testosterone, and libido following bariatric surgery. J Family Med Prim Care. (2023) 12:2596–601. doi: 10.4103/jfmpc.jfmpc_413_23, 38186840 PMC10771172

[40] Emami MR Safabakhsh M Khorshidi M Moghaddam OM Mohammed SH Zarezadeh M . Effect of bariatric surgery on endogenous sex hormones and sex hormone-binding globulin levels: a systematic review and meta-analysis. Surg Obes Relat Dis. (2021) 17:1621–36. doi: 10.1016/j.soard.2021.05.003, 34187743

[41] Puia D Ivanuta M Pricop C. Effect of bariatric surgery on male infertility: an updated meta-analysis and literature review. World J Mens Health. (2025) 43:807–17. doi: 10.5534/wjmh.240147, 40263958 PMC12505486

[42] Fallah A Mohammad-Hasani A Colagar AH. Zinc is an essential element for male fertility: a review of Zn roles in men’s health, germination, sperm quality, and fertilization. J Reprod Infertil. (2018) 19:69–81. 30009140 PMC6010824

[43] Qazi IH Angel C Yang H Zoidis E Pan B Wu Z . Role of selenium and selenoproteins in male reproductive function: a review of past and present evidences. Antioxidants. (2019) 8:268. doi: 10.3390/antiox8080268, 31382427 PMC6719970

[44] Yuan HF Zhao K Zang Y Liu CY Hu ZY Wei JJ . Effect of folate deficiency on promoter methylation and gene expression of Esr1, Cav1, and Elavl1, and its influence on spermatogenesis. Oncotarget. (2017) 8:24130–41. doi: 10.18632/oncotarget.15731, 28445960 PMC5421833

[45] Banks N Sun F Krawetz SA Coward RM Masson P Smith JF . Male vitamin D status and male factor infertility. Fertil Steril. (2021) 116:973–9. doi: 10.1016/j.fertnstert.2021.06.035, 34289935 PMC8561776

[46] Doostabadi MR Hassanzadeh-Taheri M Asgharzadeh M Mohammadzadeh M. Protective effect of vitamin E on sperm parameters, chromatin quality, and DNA fragmentation in mice treated with different doses of ethanol: an experimental study. Int J Reprod Biomed. (2021) 19:525–36. doi: 10.18502/ijrm.v19i6.9374, 34401647 PMC8350852

[47] Lauti M Kularatna M Hill AG MacCormick AD. Weight regain following sleeve gastrectomy-a systematic review. Obes Surg. (2016) 26:1326–34. doi: 10.1007/s11695-016-2152-x, 27048439

[48] Reis MG Moreira LFGG de Andra Carvalho LSV de Castro CT Vieira RAL Guimarães NS. Weight regain after bariatric surgery: a systematic review and meta-analysis of observational studies. Obes Med. (2024) 45:100528. doi: 10.1016/j.obmed.2023.100528

[49] Pham NH Bena J Bhatt DL Kennedy L Schauer PR Kashyap SR. Increased free testosterone levels in men with uncontrolled type 2 diabetes five years after randomization to bariatric surgery. Obes Surg. (2018) 28:277–80. doi: 10.1007/s11695-017-2881-5, 29143290

[50] Dhindsa S Ghanim H Jenkins T Inge TH Harmon CM Ghoshal A . High prevalence of subnormal testosterone in obese adolescent males: reversal with bariatric surgery. Eur J Endocrinol. (2022) 186:319–27. doi: 10.1530/EJE-21-0545, 35007209

[51] Gokalp F Koras O Ugur M Yildirak E Sigva H Porgali SB . Bariatric surgery has positive effects on patients’ and their partners’ sexual function: a prospective study. Andrology. (2021) 9:1119–25. doi: 10.1111/andr.13000, 33686805

