# Supplementary material for: Metabolic and bariatric surgery and male endocrine and reproductive health: a GRADE-assessed meta-analysis
Source: Front Nutr. 2026 Mar 16;13:1775651. doi: 10.3389/fnut.2026.1775651 (PMC13033656; doi:10.3389/fnut.2026.1775651)
Supplement: Supplementary file 1 [file Supplementary_file_1.docx]

**Supplementary Table 1.** Search strategies.

**PubMed**

| Search number | Query |
| --- | --- |
| 1 | Bariatric Surgery[MeSH Terms] |
| 2 | "bariatric operation*"[Title/Abstract] OR "bariatric procedure"[Title/Abstract] OR "bariatric procedures"[Title/Abstract] OR "Bariatric Surger*"[Title/Abstract] OR "Bariatric Surgical Procedure"[Title/Abstract] OR "Bariatric Surgical Procedures"[Title/Abstract] OR "Metabolic Surgeries"[Title/Abstract] OR "Metabolic Surgery"[Title/Abstract] OR "obesity operation"[Title/Abstract] OR "obesity surgery"[Title/Abstract] OR "obesity surgical treatment"[Title/Abstract] OR "Stomach Stapling"[Title/Abstract] OR "weight loss operation"[Title/Abstract] OR "weight loss surgery"[Title/Abstract] OR "weight reduction operation"[Title/Abstract] OR "weight reduction surgery"[Title/Abstract] |
| 3 | "Erectile Function Index"[Title/Abstract] OR "gonadal hormone"[Title/Abstract] OR "gonadal hormones"[Title/Abstract] OR "Gonadal Steroid Hormone"[Title/Abstract] OR "Gonadal Steroid Hormones"[Title/Abstract] OR "IIEF"[Title/Abstract] OR "IIEF International Index of Erectile Function"[Title/Abstract] OR "International Index of Erectile Function"[Title/Abstract] OR "Semen Analyses"[Title/Abstract] OR "Semen Analysis"[Title/Abstract] OR "Semen Parameter"[Title/Abstract] OR "Semen Qualities"[Title/Abstract] OR "Semen Quality"[Title/Abstract] OR "Semen Quality Analyses"[Title/Abstract] OR "Semen Quality Analysis"[Title/Abstract] OR "seminal plasma analysis"[Title/Abstract] OR "seminal quality"[Title/Abstract] OR "Sex Hormone"[Title/Abstract] OR "Sex Hormones"[Title/Abstract] OR "sex steroid"[Title/Abstract] OR "sex steroid hormone"[Title/Abstract] OR "Sex Steroid Hormones"[Title/Abstract] OR "Sexual function"[Title/Abstract] OR "sexual hormone"[Title/Abstract] OR "sperm analysis"[Title/Abstract] OR "Sperm quality"[Title/Abstract] OR "spermatic quality"[Title/Abstract] OR "spermatozoa quality"[Title/Abstract] OR "spermatozoal quality"[Title/Abstract] OR "spermatozoan quality"[Title/Abstract] OR "spermatozoid quality"[Title/Abstract] OR "spermatozoon quality"[Title/Abstract] |

**Web of Science**

| Search number | Query |
| --- | --- |
| 1 | (bariatric operation*) OR (bariatric procedure) OR (bariatric procedures) OR (Bariatric Surger*) OR (Bariatric Surgical Procedure) OR (Bariatric Surgical Procedures) OR (Metabolic Surgeries) OR (Metabolic Surgery) OR (obesity operation) OR (obesity surgery) OR (obesity surgical treatment) OR (Stomach Stapling) OR (weight loss operation) OR (weight loss surgery) OR (weight reduction operation) OR (weight reduction surgery) (Topic) and Preprint Citation Index (Exclude – Database) |
| 2 | TS=((Erectile Function Index) OR (gonadal hormone) OR (gonadal hormones) OR (Gonadal Steroid Hormone) OR (Gonadal Steroid Hormones) OR (IIEF) OR (IIEF International Index of Erectile Function) OR (International Index of Erectile Functio) OR (International Index of Erectile Function) OR (Semen Analyses) OR (Semen Analysis) OR (Semen Parameter) OR (Semen Qualities) OR (Semen Quality) OR (Semen Quality Analyses) OR (Semen Quality Analysis) OR (seminal plasma analysis) OR (seminal quality) OR (Sex Hormone) OR (Sex Hormones) OR (sex steroid) OR (sex steroid hormone) OR (Sex Steroid Hormones) OR (Sexual function) OR (sexual hormone) OR (sperm analysis) OR (Sperm quality) OR (spermatic quality) OR (spermatid quality) OR (spermatozoa quality) OR (spermatozoal quality) OR (spermatozoan quality) OR (spermatozoid quality) OR (spermatozoon quality)) and Preprint Citation Index (Exclude – Database) |

**Embase**

| Search number | Query |
| --- | --- |
| 1 | 'bariatric surgery'/exp |
| 2 | 'bariatric operation*':ab,ti,kw OR 'bariatric procedure':ab,ti,kw OR 'bariatric procedures':ab,ti,kw OR 'bariatric surger*':ab,ti,kw OR 'bariatric surgical procedure':ab,ti,kw OR 'bariatric surgical procedures':ab,ti,kw OR 'metabolic surgeries':ab,ti,kw OR 'metabolic surgery':ab,ti,kw OR 'obesity operation':ab,ti,kw OR 'obesity surgery':ab,ti,kw OR 'obesity surgical treatment':ab,ti,kw OR 'stomach stapling':ab,ti,kw OR 'weight loss operation':ab,ti,kw OR 'weight loss surgery':ab,ti,kw OR 'weight reduction operation':ab,ti,kw OR 'weight reduction surgery':ab,ti,kw |
| 3 | 'erectile function index':ab,ti,kw OR 'gonadal hormone':ab,ti,kw OR 'gonadal hormones':ab,ti,kw OR 'gonadal steroid hormone':ab,ti,kw OR 'gonadal steroid hormones':ab,ti,kw OR 'iief':ab,ti,kw OR 'iief international index of erectile function':ab,ti,kw OR 'international index of erectile functio':ab,ti,kw OR 'international index of erectile function':ab,ti,kw OR 'semen analyses':ab,ti,kw OR 'semen analysis':ab,ti,kw OR 'semen parameter':ab,ti,kw OR 'semen qualities':ab,ti,kw OR 'semen quality':ab,ti,kw OR 'semen quality analyses':ab,ti,kw OR 'semen quality analysis':ab,ti,kw OR 'seminal plasma analysis':ab,ti,kw OR 'seminal quality':ab,ti,kw OR 'sex hormone':ab,ti,kw OR 'sex hormones':ab,ti,kw OR 'sex steroid':ab,ti,kw OR 'sex steroid hormone':ab,ti,kw OR 'sex steroid hormones':ab,ti,kw OR 'sexual function':ab,ti,kw OR 'sexual hormone':ab,ti,kw OR 'sperm analysis':ab,ti,kw OR 'sperm quality':ab,ti,kw OR 'spermatic quality':ab,ti,kw OR 'spermatid quality':ab,ti,kw OR 'spermatozoa quality':ab,ti,kw OR 'spermatozoal quality':ab,ti,kw OR 'spermatozoan quality':ab,ti,kw OR 'spermatozoid quality':ab,ti,kw OR 'spermatozoon quality':ab,ti,kw |

**Cochrane**

| Search number | Query |
| --- | --- |
| 1 | MeSH descriptor: [Bariatric Surgery] explode all trees |
| 2 | ('bariatric operation*' OR 'bariatric procedure' OR 'bariatric procedures' OR 'Bariatric Surger*' OR 'Bariatric Surgical Procedure' OR 'Bariatric Surgical Procedures' OR 'Metabolic Surgeries' OR 'Metabolic Surgery' OR 'obesity operation' OR 'obesity surgery' OR 'obesity surgical treatment' OR 'Stomach Stapling' OR 'weight loss operation' OR 'weight loss surgery' OR 'weight reduction operation' OR 'weight reduction surgery'):ab,ti,kw |
| 3 | ('Erectile Function Index' OR 'gonadal hormone' OR 'gonadal hormones' OR 'Gonadal Steroid Hormone' OR 'Gonadal Steroid Hormones' OR 'IIEF' OR 'IIEF International Index of Erectile Function' OR 'International Index of Erectile Functio' OR 'International Index of Erectile Function' OR 'Semen Analyses' OR 'Semen Analysis' OR 'Semen Parameter' OR 'Semen Qualities' OR 'Semen Quality' OR 'Semen Quality Analyses' OR 'Semen Quality Analysis' OR 'seminal plasma analysis' OR 'seminal quality' OR 'Sex Hormone' OR 'Sex Hormones' OR 'sex steroid' OR 'sex steroid hormone' OR 'Sex Steroid Hormones' OR 'Sexual function' OR 'sexual hormone' OR 'sperm analysis' OR 'Sperm quality' OR 'spermatic quality' OR 'spermatid quality' OR 'spermatozoa quality' OR 'spermatozoal quality' OR 'spermatozoan quality' OR 'spermatozoid quality' OR 'spermatozoon quality'):ab,ti,kw |

**Supplementary Figure 1-14.** Funnel plots for all included values at 3 postoperative intervals.


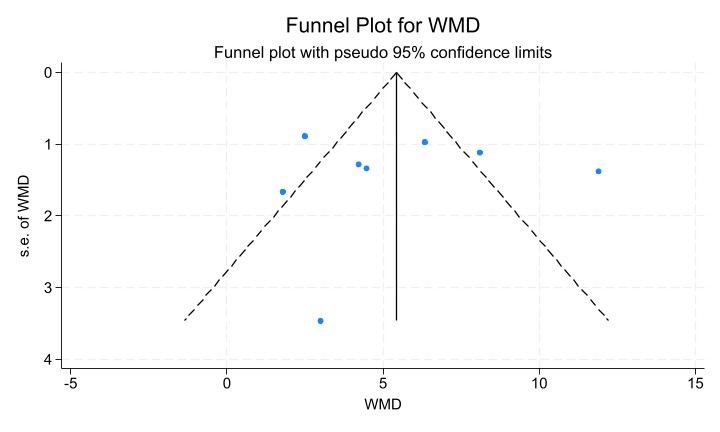


1. TT at 1-3 M


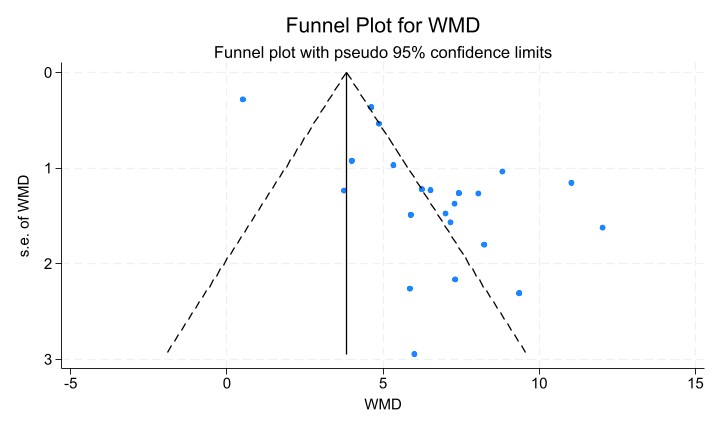


1. TT at 6-9 M


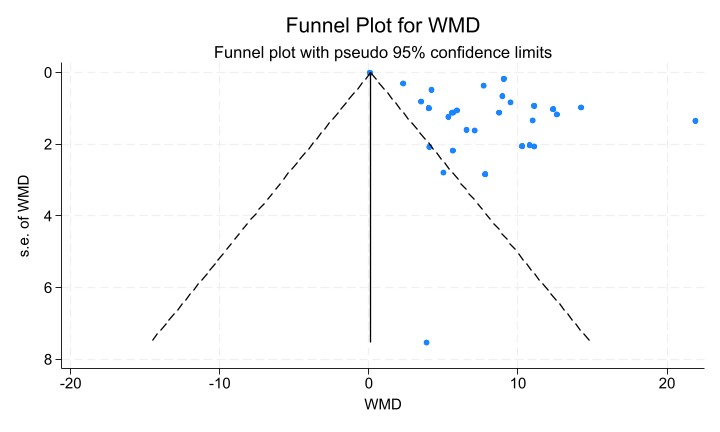


1. TT at >12 M


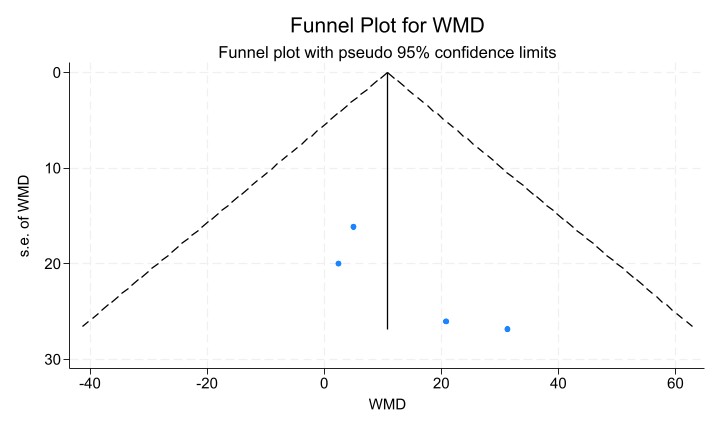


1. FT at 1-3 M


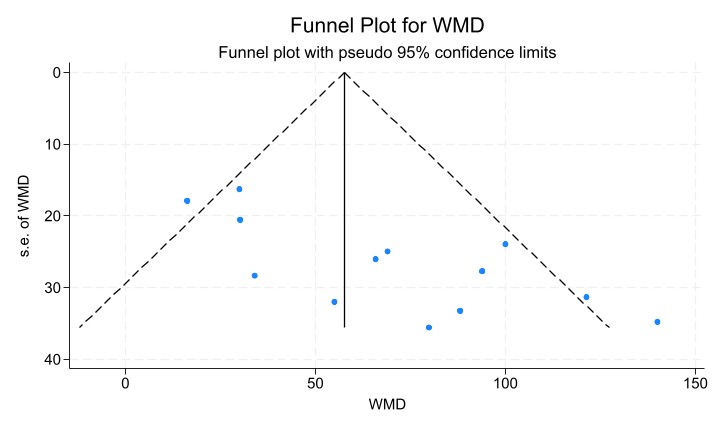


1. FT at 6-9 M


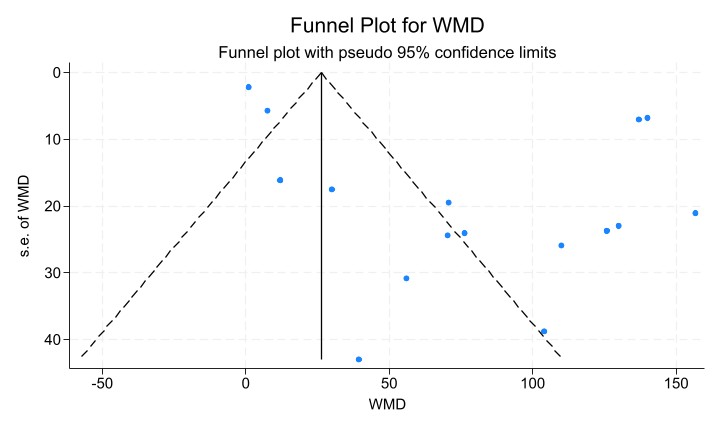


1. FT at >12 M


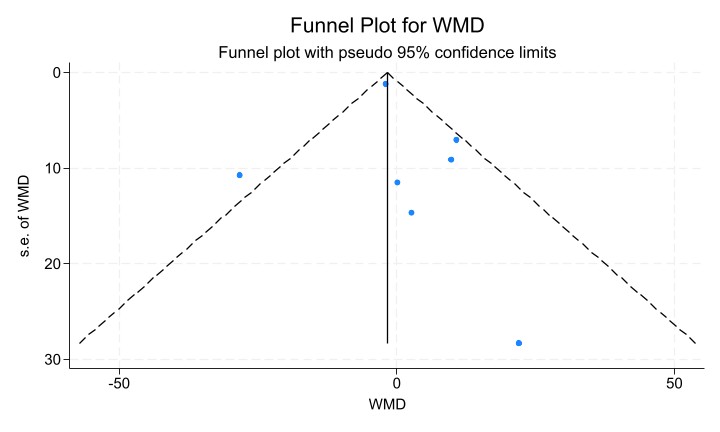


1. E_2_ at 1-3 M


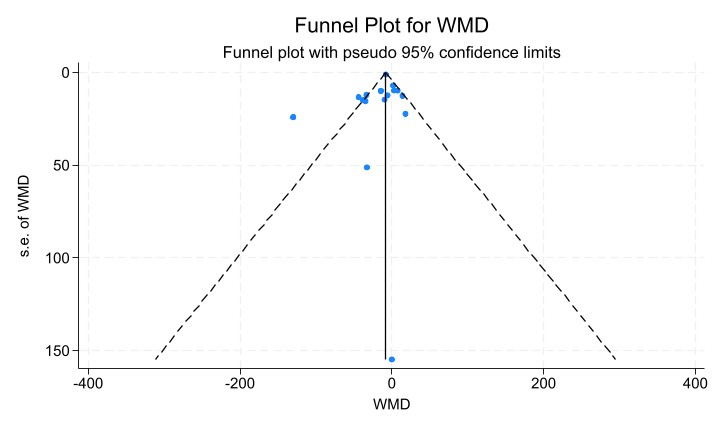


1. E_2_ at 6-9 M


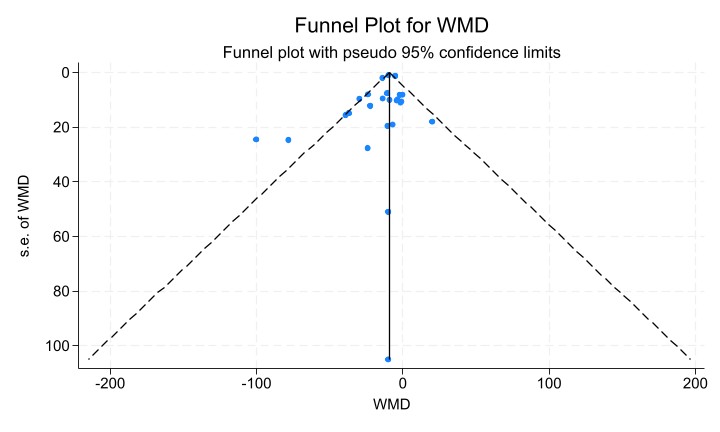


1. E_2_ at >12 M


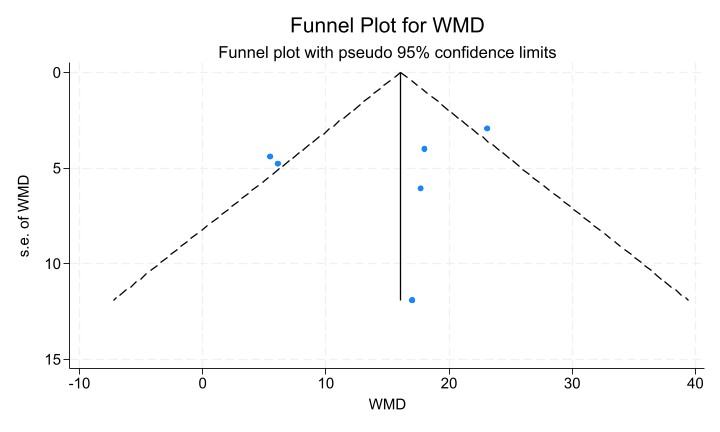


1. SHBG at 1-3 M


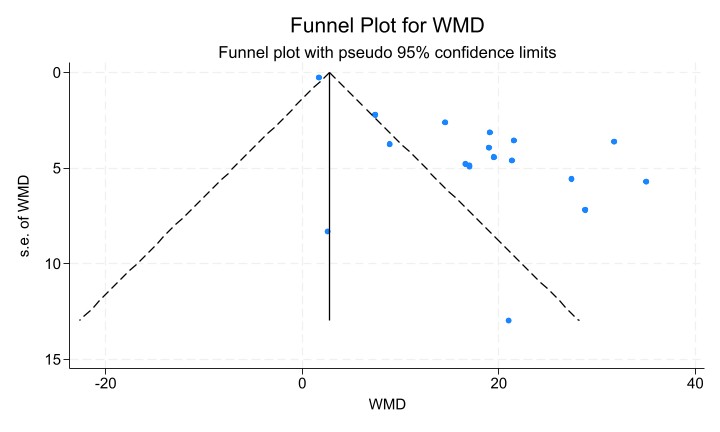


1. SHBG at 6-9 M


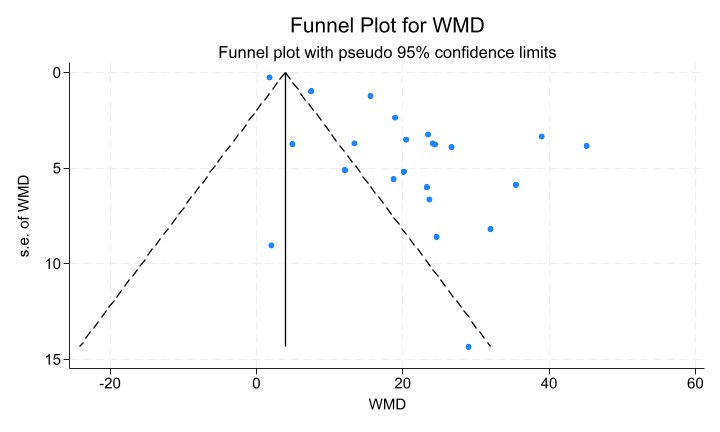


1. SHBG at > 12M


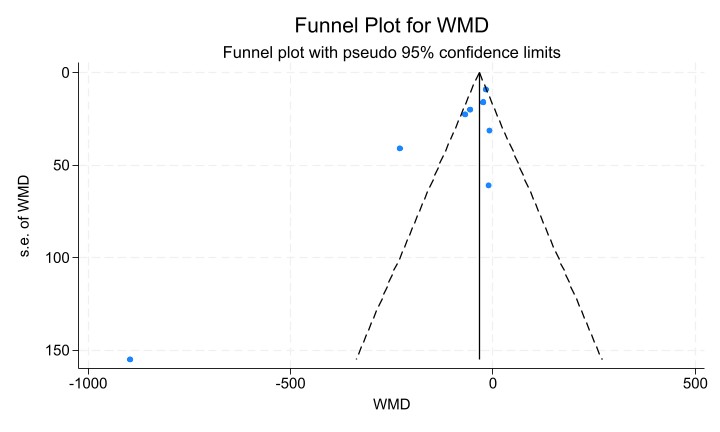


1. PRL


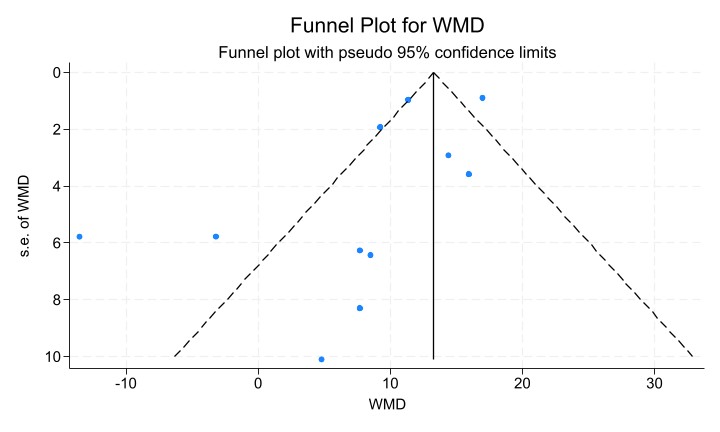


1. IIEF
